# Polygenic Risks for Mood Disorders and Economic Well‐being: Study of Finnish Cohorts

**DOI:** 10.1155/da/1008569

**Published:** 2026-02-23

**Authors:** Aaro Hazak, Johanna Liuhanen, Katri Kantojärvi, Merike Kukk, Sonja Sulkava, Tuija Jääskeläinen, Veikko Salomaa, Seppo Koskinen, Markus Perola, Tiina Paunio

**Affiliations:** ^1^ Department of Public Health, Finnish Institute for Health and Welfare, Helsinki, Finland, thl.fi; ^2^ Department of Psychiatry/SleepWell Research Program, Faculty of Medicine, University of Helsinki, Helsinki, Finland, helsinki.fi; ^3^ Department of Finance, Aalto University, Espoo, Finland, aalto.fi; ^4^ Department of Economics and Finance, Tallinn University of Technology, Tallinn, Estonia, ttu.ee; ^5^ Department of Psychiatry, Helsinki University Hospital, Helsinki, Finland, hus.fi; ^6^ Department of Clinical Genetics, Helsinki University Hospital, Helsinki, Finland, hus.fi; ^7^ Department of Internal Medicine, University of Turku, Turku, Finland, utu.fi; ^8^ Research Program for Clinical and Molecular Metabolism, Faculty of Medicine, University of Helsinki, Helsinki, Finland, helsinki.fi

**Keywords:** bipolar disorder, depression, employment, income, occupation, polygenic score, satisfaction

## Abstract

**Background:**

Polygenic scores (PGS) for mood disorders provide population‐level measures of genetic liability, allowing examination of how common mental health‐related traits associate with socio‐economic outcomes. This study investigated how PGS for depression (DPGS), bipolar disorder (BDPGS) and overall mood disorders (MDPGS) predict economic outcomes in the general population.

**Methods:**

We studied genetic and socio‐economic registry data alongside repeated cross‐sectional surveys from six Finnish cohorts (1992–2017; *N* = 20,121; ages 25–64), representative of various regions. Using multiple regression models, we examined associations between PGS and educational attainment, employment status, occupational type, equivalent income and economic satisfaction.

**Results:**

All PGS were negatively associated with employment probability, although their associations with other economic outcomes varied depending on educational attainment as a mediating factor. BDPGS was positively associated with higher educational attainment and engagement in knowledge work, particularly among females. However, BDPGS showed no significant associations with equivalent income or economic satisfaction. In contrast, DPGS was negatively associated with educational attainment and demonstrated negative associations with knowledge work, equivalent income and economic satisfaction. MDPGS, consolidating depression and bipolar disorder (BD) risks, showed no significant association with educational attainment but was negatively associated with equivalent income and economic satisfaction. Additionally, DPGS and MDPGS were linked to a lower likelihood of self‐employment among males.

**Conclusions:**

The genetic predispositions for depression and BD exhibit distinct and sometimes opposing relationships with economic outcomes, mediated by education. Although effect sizes were substantial, genetic risks could still be mitigated by environmental factors, such as education and institutional frameworks, that foster economic resilience. The lack of association between MDPGS and educational level highlights the offsetting effects of its components, suggesting that focusing on specific mental disorders rather than generalisations offers clearer insights into the genetic underpinnings of brain health‐related economic disparities in the general population.

## 1. Introduction

Mood disorders, including depression and bipolar disorder (BD), represent major global public health challenges characterised by impaired psychosocial functioning, substantial personal suffering and profound societal implications [1]. These conditions generate considerable economic burden through reduced productivity, lower economic attainment and increased reliance on healthcare and social services [2–6]. However, mood disorder‐related traits exist on a continuum across the population rather than being limited to clinical diagnoses. Understanding how these widely shared mental health predispositions relate to social and economic outcomes can help inform supportive environments that promote inclusive opportunities for diverse population groups and reduce stigma by framing vulnerability as a normal aspect of human variation.

Major depressive disorder (MDD) affects ~4.4% of the global population annually, with lifetime prevalence estimates reaching up to 20% [7–9]. It is about twice as common in women as in men, a pattern consistently observed across cultures and diagnostic criteria [10, 11]. Twin studies estimate its heritability at 30%–40%, while genome‐wide data indicate single‐nucleotide polymorphism (SNP)‐based heritability of 9%–10% [12, 13]. BD affects ~0.6%–1% of the population worldwide [14, 15], with heritability estimates ranging from 60% to 85% in twin studies [16, 17] and SNP‐heritability around 19%–22% [12, 18, 19]. Although some studies report slightly higher prevalence among women, no consistent sex differences in heritability have been established [17, 20].

Polygenic scores (PGS)—composite measures capturing the additive effects of common genetic variants—offer a means to quantify inherited liability for complex traits such as depression (DPGS) [21], bipolar disorder (BDPGS) [18] and a combined score for these two mood disorders (MDPGS) [22]. While PGS do not identify specific biological mechanisms or predict individual outcomes, they provide population‐level indicators of shared genetic influences that can help clarify how genetic liability manifests in diverse life domains. Examining such associations in the general population—not only among clinically diagnosed individuals—provides valuable insight into the broader distribution of mental health‐related traits and their societal correlates.

Previous studies have reported complex and sometimes contradictory genetic correlations between mood disorder risk and socio‐economic outcomes. For BD, a weak positive genetic correlation has been found with educational attainment (*rg* = 0.11) [23], contrasting with a weak negative genetic correlation with intelligence (*rg* = −0.09) [18]. This complexity is further reflected in mixed findings linking BD to both low and high intelligence and school grades [24–26], as well as negative associations with cognitive functioning in children with elevated BDPGS [27]. These findings suggest a nuanced relationship between BD polygenic risk and the quantity and quality of education, as well as cognitive abilities. Furthermore, BD demonstrates weak to moderate genetic correlations with various socio‐economic indicators, including socio‐economic status (*rg* = 0.13), inability to work (*rg* = 0.23), income (*rg* = 0.07), financial situation satisfaction (*rg* = 0.10) and subjective well‐being (*rg* = −0.25) [18, 23, 28]. These contradictory associations underscore the complex interplay between BD genetic risk and socio‐economic outcomes, suggesting that BD‐related traits may both facilitate and hinder individual success across different domains.

Depression, in turn, exhibits substantial negative genetic correlations with both educational and economic outcomes, encompassing educational attainment (*rg* = −0.19), college completion (*rg* = −0.19), years of schooling (*rg* = −0.15), socio‐economic status (*rg* = −0.42), income (*rg* = −0.24) and subjective well‐being (*rg* = −0.64) [21, 23, 28]. These findings indicate that shared genetic influences may predispose to both poorer mental health and less favourable socio‐economic outcomes.

Leveraging data from Finnish general population cohorts spanning 25 years, this study comparatively investigates the relationship between polygenic risks for mood disorders and key socio‐economic indicators: educational attainment, employment, occupation, income and subjective economic well‐being. The progression from education to objective and subjective economic well‐being represents a sequential pathway where education shapes employment opportunities and occupational choices, which in turn influence income levels [29–31]. Higher income contributes to improved economic satisfaction by enhancing financial stability and access to resources, completing the link between foundational education and overall economic well‐being [32]. Finland provides an informative setting, with universal education and healthcare and relatively low income inequality, allowing investigation of these associations within a socially supportive context. Our focus is not on identifying biological mechanisms or assigning causality, but on characterising population‐level patterns that reflect the interplay between inherited mental health vulnerability and socio‐economic opportunity. Understanding these links can inform both research and practice by identifying social environments that may help to buffer genetic disadvantage and promote resilience, while countering deterministic or stigmatising interpretations of genetic influence.

## 2. Methods

### 2.1. Study Cohorts

The study combined genetic and registry data with repeated cross‐sectional surveys from the Finnish National FINRISK Study (FR; five pentennial cohorts spanning 1992–2012) [33] and the FinHealth 2017 Study (FH) [34], both coordinated by the Finnish Institute for Health and Welfare (THL). These surveys represent independent, regionally stratified random samples of the Finnish adult population across major regions, with field centres in Helsinki and Vantaa (Southern Finland), Turku and Loimaa (Southwest), Tampere (Western), Kuopio and Joensuu (Eastern), Oulu (Northern) and Lahti (Southern Finland, FH cohort only), conducted according to standardised protocols [33, 34].

Participants aged 25–64 years were included, with additional criteria ensuring more precise income measurement: households with a maximum of 10 members and no more than one additional adult besides the respondent. After applying exclusion criteria—including age range limitations, missing genetic or registry data, residents of Lapland (data not available for most study cohorts) and cases of inconsistent household reporting—the final pooled 1992–2017 sample comprised 20,121 participants. The participant flowchart is presented in supplementary material of [35]. The average age of participants in the sample was 44.9 years, and 46% were males, with age and gender distributions comparable across the six study cohorts [33, 34].

### 2.2. Genetic Measures

The primary explanatory variables were DPGS, BDPGS and MDPGS, derived from blood samples collected during on‐site visits of participants in the FR and FH cohorts. DPGS was constructed using data from a GWAS meta‐analysis of 173,005 individuals of European ancestry from the Psychiatric Genomics Consortium (PGC), where depression was identified through self‐reported medical treatment or diagnosis [21]. BDPGS was based on a GWAS of 413,366 individuals of European ancestry from the PGC, identifying cases via expert assessment or medical records of lifetime BD diagnosis [18]. MDPGS combined data from a GWAS of 296,612 individuals of European ancestry from the PGC, defining cases as having a lifetime diagnosis of either MDD or BD [22]. We employed PRS‐CS Bayesian regression to estimate posterior variant effects for constructing DPGS, BDPGS and MDPGS, using European 1000 Genomes Project samples as the reference population. Final scores were calculated as the weighted sum of risk allele counts for 1,085,713 (DPGS), 1,097,131 (BDPGS) and 984,561 (MDPGS) variants, standardised to a mean of 0 and standard deviation of 1. No individual‐level covariates were included in the PGS computation; variables such as gender, age and principal components were incorporated only in the subsequent regression analyses. PGS were generated using the PRS‐CS‐auto method [36], which infers the global shrinkage parameter (*φ*) directly from the data and therefore does not require manual specification. Participants with missing genetic data were excluded prior to analysis, resulting in complete‐case analyses for all models. Genotyping was performed separately for the FH and FR cohorts, with minimal overlap between FH participants and the 5 FR cohorts. Details of the genotyping methodology are provided in Supplementary Material of [35].

In our sample, Wilcoxon rank‐sum tests compared the distributions of DPGS, BDPGS and MDPGS between two groups: individuals with a recorded diagnosis of the respective mental disorders and those without. The tests revealed highly significant differences for all three PGSs (*p* < 0.001), indicating that these PGS effectively differentiate between individuals with and without the disorders. The mental disorder prevalence was determined using health registry data, identifying individuals who had received clinical inpatient treatment for these conditions by the study year. Diagnoses were based on the 10^th^ edition of the International Classification of Diseases (ICD‐10) or corresponding diagnoses from earlier ICD editions: manic episode (F30) and bipolar disorder (F31) for BD and depressive episode (F32) and recurrent depressive disorder (F33) for depression.

### 2.3. Outcome Measures

The study examined five interconnected economic well‐being outcomes following a clear sequential progression: education, employment, occupation, equivalent income and economic satisfaction.

Educational attainment (primary, secondary or higher) was obtained from Statistics Finland registry data. Employment status and occupation were derived from Statistics Finland labour market status registry data, categorised as non‐employed, self‐employed, physical work, office work or knowledge work and measured at the beginning of each study year (with exceptions for the 1992, 1997 and 2002 cohorts, where data was from the preceding year). Since education and occupational status rarely change in adulthood, and genetic proxies for mood disorders remain constant throughout life, the repeated cross‐sectional design of our data does not pose significant limitations to this study.

Equivalent income was assessed using self‐reported household income brackets, converted to continuous variables and adjusted by adult household members using the OECD equivalence scale (primary adult: 1.0, other adults: 0.5 and children: 0.3) [37], then translated into comparable cross‐year tertiles. This approach is supported by evidence of assortative marriage patterns in Nordic countries, where partners typically have comparable educational and economic backgrounds [38]. Tertiles enable comparisons among the highest, medium and lowest equivalent income groups, recognising that the self‐reported household income, provided in range categories, does not allow for high‐granularity analysis.

Satisfaction with their economic situation was measured through self‐reported responses, dichotomised into satisfied (very satisfied/satisfied/somewhat satisfied) versus unsatisfied (unsatisfied/very unsatisfied) categories.

### 2.4. Control Measures

Control variables included gender, age and birth cohort, sourced from the Finnish National Population Register, which provided reliable and exogenous demographic indicators. Potentially endogenous socio‐economic control variables, while relevant for determining economic outcomes, were excluded to avoid attenuating the total association between genetic proxies for mood disorders and economic outcomes.

To account for population stratification, the first three principal components (PC1–3) of the genetic data were included as controls. This approach aligns with established practices in studies using Finnish samples, reflecting the genetic homogeneity of the Finnish population. For sensitivity analysis, we also present the main results from comparable models incorporating PC1–10.

Additionally, study year dummies were incorporated into the regression models to capture cohort‐specific effects and to adjust for temporal variations inherent to the repeated cross‐sectional study design.

### 2.5. Descriptive Statistics

Table 1 presents descriptive statistics of the pooled 1992–2017 sample, Supporting information 1: Figure S1 histograms of DPGS, BDPGS and MDPGS by gender, and Supporting Information 1: Table S1 the pairwise linear correlation matrix of study variables.

**Table 1:** Descriptive statistics of the pooled 1992–2017 sample.

Variable	Explanation	All		Male		Female		Lowest decile DPGS^a^		Highest decile DPGS^a^		Lowest decile BDPGS^a^		Highest decile BDPGS^a^		Lowest decile MDPGS^a^		Highest decile MDPGS^a^	
		Mean (%)	Min– Max (SD)	Mean (%)	Min– Max (SD)	Mean (%)	Min– Max (SD)	Mean (%)	Min– Max (SD)	Mean (%)	Min– Max (SD)	Mean (%)	Min– Max (SD)	Mean (%)	Min– Max (SD)	Mean (%)	Min– Max (SD)	Mean (%)	Min– Max (SD)
*N*	—	20,121	—	9248	—	10,873	—	1998	—	1995	—	2022	—	2017	—	1981	—	1998	—
DPGS	—	−0.006	−4.006– 4.040 (0.998)	−0.019	−4.006– 4.040 (1.000)	0.004	−3.958– 3.906 (0.995)	−1.774	‐4.006– −1.300 (0.412)	1.737	1.270– 4.040 (0.422)	−0.548	−3.976– 2.966 (0.998)	0.419	−2.490– 3.906 (0.941)	−1.253	−4.006– 1.174 (0.774)	1.230	−1.030– 4.040 (0.762)
BDPGS	—	−0.009	−3.696– 4.227 (1.003)	−0.023	−3.696– 4.227 (1.005)	0.003	−3.617– 3.878 (1.002)	−0.548	−3.696– 2.541 (1.007)	0.442	−2.945– 4.227 (0.944)	−1.799	−3.696– −1.301 (0.432)	1.722	1.255– 4.227 (0.405)	−1.033	−3.696– 2.328 (0.903)	0.931	−2.316– 4.227 (0.868)
MDPGS	—	−0.001	−3.885– 3.898 (0.998)	−0.014	−3.885– 3.898 (1.003)	0.010	−3.589– 3.486 (0.993)	−1.267	−3.885– 1.085 (0.749)	1.235	−1.113– 3.898 (0.740)	−1.024	−3.885– 2.304 (0.874)	0.922	−1.622– 3.898 (0.858)	−1.773	−3.885– −1.300 (0.409)	1.740	0.401– 1.275 (3.898)
Education																			
1	Primary (ref)	20.2%	—	22.1%	—	18.5%	—	17.5%	—	22.9%	—	20.4%	—	21.9%	—	19.5%	—	21.1%	—
2	Secondary	42.1%		44.9%		39.8%		40.9%		42.8%		43.7%		40.1%		41.7%		42.2%	
3	Higher	37.7%		33.0%		41.7%		41.6%		34.3%		36.0%		38.1%		38.8%		36.6%	
Labour market status^b^																			
1	Non‐employed	24.6%	—	26.3%	—	23.1%	—	21.0%	—	29.1%	—	21.8%	—	28.2%	—	20.7%	—	28.9%	—
2	Self‐employed	9.0%		11.6%		6.7%		10.0%		7.9%		9.0%		9.1%		10.2%		8.8%	
3	Physical work	21.1%		27.6%		15.6%		21.4%		20.6%		24.4%		19.3%		22.3%		19.7%	
4	Office work	28.0%		16.8%		37.6%		28.3%		29.1%		29.1%		25.5%		28.4%		26.7%	
5	Knowledge work	17.3%		17.8%		16.9%		19.4%		13.3%		15.7%		17.9%		18.4%		15.9%	
Equivalent income tertile^c^																			
1	Lowest	33.2%	—	31.9%	—	34.2%	—	31.2%	—	36.8%	—	31.8%	—	36.6%	—	29.3%	—	35.6%	—
2	Medium	34.9%		33.7%		36.0%		34.8%		34.9%		35.8%		33.8%		36.4%		35.0%	
3	Highest	31.9%		34.4%		29.7%		34.1%		28.4%		32.3%		29.6%		34.3%		29.3%	
Economic satisfaction	Satisfied = 1	82.6%	—	81.7%	—	83.4%	—	85.2%	—	80.5%	—	81.8%	—	81.8%	—	85.6%	—	80.9%	—
Gender	Male = 0 (ref)	46.0%	—	100%	—	0%	—	47.7%	—	44.3%	—	47.3%	—	45.8%	—	47.4%	—	46.4%	—
Age		44.9	25–64 (11.7)	45.3	25–64 (11.6)	44.5	25–64 (11.7)	44.4	25–64 (11.8)	45.1	25–64 (11.7)	44.6	25–64 (11.6)	45.0	25–64 (11.6)	44.5	25–64 (11.7)	45.1	25–64 (11.7)

^a^All sample members in the lowest decile or highest decile of each polygenic score (PGS): depression (DPGS) [21], bipolar disorder (BDPGS) [18] and mood disorders (MDPGS) [22].

^b^‘Labour market status’ encompasses both employment status (designated as category 1 ‘Non‐employed’) and occupational categories (categories 2–5). Specifically, category 2, ‘Self‐employed’, includes individuals classified as ‘Self‐employed persons’; category 3, ‘Physical work’, comprises those classified as ‘Manual workers’; category 4, ‘Office work’, includes individuals classified as ‘Lower‐level employees with administrative and clerical occupations’; and category 5, ‘Knowledge work’, encompasses those classified as ‘Upper‐level employees with administrative, managerial, professional and related occupations’ according to the Statistics Finland registry data.

^c^Some equivalent income tertile sizes in the full sample differ slightly from 1/3 because income was measured in range categories in the FR and FH surveys, which does not allow for allocation of participants into income groups of exactly 33.3%.

### 2.6. Statistical Analysis

The research employed multiple regression approaches to examine relationships between PGS (DPGS, BDPGS and MDPGS) and economic well‐being outcomes in their sequential progression. Educational attainment was examined using ordered probit regression models, incorporating PGS as a key explanatory variable, alongside controls for gender, birth cohort dummies and genetic principal components (PC1–3).

Labour market status was examined using a two‐stage approach. First, probit models were employed to assess the total associations of DPGS, BDPGS and MDPGS with labour market status. These models included PGS as the primary explanatory variable and controlled for gender, age (in both linear and squared terms), birth cohort dummies, study year dummies and PC1–3. Second, recognising that genetic risks for mood disorders may be associated with the likelihood of attaining higher levels of education, we accounted for the possibility that genetic risk factors relate to income both through education and through other pathways. To disentangle the mediator role of education from other channels, ordered probit models were estimated separately within three educational level subgroups. This stratified approach allows the examination of associations between PGS and labour market status beyond differences between educational levels. These effects likely reflect cognitive and non‐cognitive skills [31], including those related to the quality of education (as distinct from the quantity captured by educational level), as well as other traits linked to the PGS. Differences in economic outcomes across educational and other categories are presented in Supporting Information 1: Figures S2–S5, reflecting well‐established links between education and economic performance [29–32], while the stratified ordered probit models reveal the associations between genetic risk and economic outcomes that extend beyond educational attainment.

Equivalent income tertiles were studied using ordered probit models to assess total associations with DPGS, BDPGS and MDPGS. Subsequently, similar models were applied within educational level subgroups to examine non‐education‐mediated associations, following the same methodology as the labour market status models.

Economic satisfaction was examined using probit models to assess total associations with DPGS, BDPGS and MDPGS. To further examine how these associations are shaped by preceding relationships with equivalent income, additional probit models were estimated within each equivalent income tertile.

All models utilised robust standard errors to account for potential heteroscedasticity, outliers, non‐normality and clustered data. Predicted probabilities for educational levels, labour market statuses, highest equivalent income tertile and economic satisfaction were calculated at different levels of DPGS, BDPGS and MDPGS. Average marginal effects represent percentage point (pp) differences in outcome probabilities compared to the lowest decile of relevant PGS as reference levels. Statistical significance was set at *p* < 0.005, with results considered suggestive at *p* < 0.05, following guidelines for multiple testing correction across subsamples (by gender, gender‐educational level and gender‐equivalent income tertile subsamples) and recommendations of Benjamin et al. [39]. In addition to presenting results for the full sample, this study highlights gender differences to account for disparities in educational attainment, occupational segregation and income (Table 1).

Analyses were conducted using STATA 18 software, maintaining methodological consistency with the research group’s other studies on genetic proxies for psychiatric and brain health traits and socio‐economic outcomes [35, 40, 41]. During the preparation of this work, the authors used Scholar GPT, a generative AI tool, in order to assist with literature searches and improve readability and language. After using these tools, the authors reviewed and edited the content as needed and take full responsibility for the content of the publication. A completed STROBE Statement checklist for observational studies is included in Supporting Information 2.

## 3. Results

### 3.1. Educational Attainment and Genetic Associations

**Figure 1:**
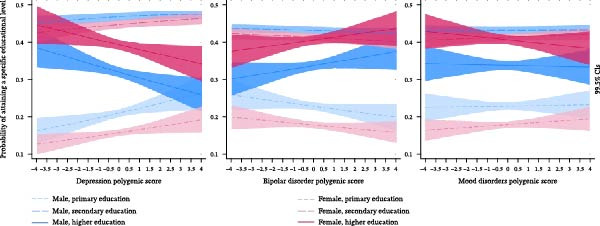
Polygenic scores for depression, bipolar disorder and mood disorders and predicted probabilities of attaining a specific educational level in the pooled 1992–2017 sample (*N* = 20,121) with 99.5% CI. Note: Figures based on predicted probabilities from ordered probit regression models of educational level, as detailed in Supporting Information 1: Table S2, using a linear specification of the polygenic score as the main explanatory variable.

We first examined the relationship between DPGS, BDPGS and MDPGS with educational attainment, a critical determinant of economic success. Using ordered probit models, DPGS was significantly negatively associated with educational attainment (*p* < 0.001), while BDPGS showed a suggestive positive association (*p* = 0.02) in the overall sample (Figure 1, Supporting Information 1: Table S2). Interaction terms between gender and both DPGS and BDPGS were non‐significant, indicating no meaningful gender moderation in the full sample. However, in separate analyses for males and females, which allows for observing gender‐specific effects of all covariates, BDPGS demonstrated a suggestive positive association with education in males (*p* = 0.02) but not in females, pointing to potential gender‐specific gene–environment correlations or interactions.

Comparing deciles, individuals in the highest DPGS decile were 6.2 pp less likely to attain higher education than those in the lowest decile (*p* < 0.001; 99.5% CI: 2.7–9.7 pp; Supporting Information 1: Table S3). For BDPGS, while the highest decile showed high variance, individuals in the 9th decile were 3.6 pp more likely to attain higher education than the lowest decile (*p* = 0.004; 99.5% CI: 0.1–7.1 pp). No significant non‐linear associations were observed for DPGS or BDPGS with educational attainment (Supporting Information 1: Table S2). MDPGS was not significantly associated with education in any model (Figure 1, Supporting Information 1: Table S2).

### 3.2. Labour Market Status and Total Genetic Associations

**Figure 2:**
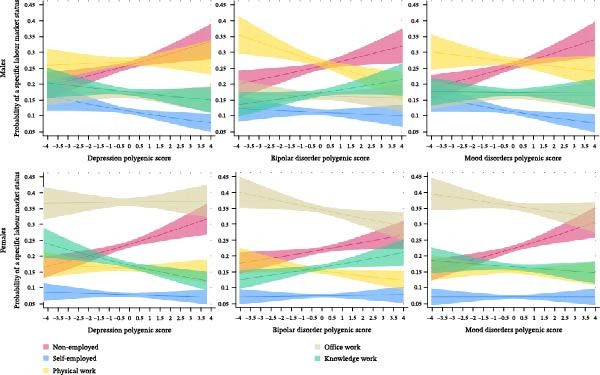
Polygenic scores for depression, bipolar disorder and mood disorders and predicted probabilities of labour market status in the pooled 1992–2017 sample (*N* = 20,121) with 99.5% CI. Note: Figures are based on predicted probabilities from probit regression models of labour market status, as detailed in Supporting Information 1: Tables S4–S9, using a linear specification of the polygenic score as the main explanatory variable.

We next assessed the total associations of the three PGS with labour market status, starting with employment. Probit models revealed significant negative associations of DPGS, BDPGS and MDPGS with employment status across genders (*p* < 0.005; Figure 2, Supporting Information 1: Tables S4–S9). Interaction terms between gender and PGSs were non‐significant, indicating no substantial gender‐based differences in the overall associations (Supporting Information 1: Tables S4, S6, S8). Compared to the lowest decile, individuals in the highest decile of DPGS, BDPGS and MDPGS were 6.9 pp (*p* <0.001; 99.5% CI: 3.3–10.5 pp), 5.5 pp (*p* < 0.001; 99.5% CI: 1.9–9.1 pp) and 7.2 pp (*p* < 0.001; 99.5% CI: 3.6–10.8 pp) less likely to be employed, respectively (Supporting Information 1: Table S10).

For occupational categories, DPGS showed no association with knowledge work in the full sample or among males but exhibited a significant negative relationship among females (*p* < 0.001; Figure 2, Supporting Information 1: Table S5). Females in the highest DPGS decile were 6.1 pp less likely (*p* < 0.001; 99.5% CI: 1.8–10.3 pp) to engage in knowledge work compared to those in the lowest decile (Supporting Information 1: Table S10). BDPGS demonstrated a suggested positive association with knowledge work across genders, with the association being particularly strong in females (*p* < 0.001; Figure 2, Supporting Information 1: Tables S6–S7). Individuals in the highest BDPGS decile were 3.3 pp more likely (*p* = 0.004; 99.5% CI: 0.1–6.5 pp) to engage in knowledge work compared to those in the lowest decile (Supporting Information 1: Table S10). MDPGS showed no association with knowledge work (Supporting Information 1: Tables S8–S9).

BDPGS was negatively associated with physical work and, among females but not in the full sample or males, with office work (*p* < 0.001; Figure 2, Supporting Information 1: Tables S6–S7). Those in the highest BDPGS decile were 4.8 pp less likely to engage in physical work (*p* < 0.001; 99.5% CI: 1.3–8.3 pp), while females in the highest decile were suggested to be 5.4 pp less likely to engage in office work (*p* = 0.008; 99.5% CI: −0.3–11.1 pp) compared to those in the lowest decile (Supporting Information 1: Table S10).

In the full sample, DPGS was negatively associated with self‐employment (*p* = 0.001) with no significant interaction by gender, though subsample analyses indicated the association was more prominent in males (*p* = 0.001) than females (Figure 2, Supporting Information 1: Tables S4–S5). MDPGS was negatively associated with self‐employment in males (*p* = 0.002; Figure 2, Supporting Information 1: Tables S8–S9). Males in the highest DPGS decile were 4.2 pp less likely (*p* = 0.004; 99.5% CI: 0.1–8.4 pp) and those in the highest MDPGS decile 3.9 pp less likely (*p* = 0.009; 99.5% CI 0.0–8.1 pp) to be self‐employed compared to the lowest decile (Supporting Information 1: Table S10). BDPGS was not significantly associated with self‐employment (Supporting Information 1: Tables S6–S7). Neither DPGS, BDPGS nor MDPGS demonstrated statistically and economically significant non‐linear associations with labour market status (Supporting Information 1: Tables S4–S9).

### 3.3. Labour Market Status: Genetic Associations Beyond Education‐Related Pathways

By examining the associations between genetic risk and labour market status within educational categories, we aimed to determine whether genetic risk factors are linked to labour market outcomes after accounting for their role in educational selection. Significant associations within educational levels would indicate pathways beyond educational attainment, such as cognitive and non‐cognitive skills, health or labour market dynamics. Conversely, non‐significant associations would suggest that the links between PGSs and labour market status are primarily mediated by educational attainment.

The probit models stratified by educational categories (Supporting Information 1: Tables S11–S13) did not reveal statistically significant associations between a PGS and specific labour market statuses within most educational categories. However, notable exceptions included negative associations of DPGS and MDPGS with self‐employment within the secondary education category (*p* < 0.005) and positive associations of non‐employment with BDPGS within the primary education category (*p* < 0.005) and with MDPGS within the secondary education category (*p* < 0.001). These findings suggest that PGSs are associated with employment and self‐employment statuses through pathways that extend beyond educational level.

### 3.4. Equivalent Income and Total Genetic Associations

**Figure 3:**
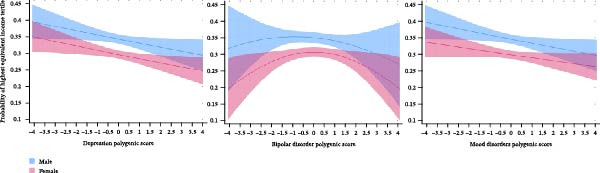
Polygenic scores for depression, bipolar disorder and mood disorders and predicted probabilities of belonging to the highest equivalent income tertile in the pooled 1992–2017 sample (*N* = 19,685) with 99.5% CI. Note: Figures using predicted probabilities from the ordered probit models of equivalent income tertiles, presented in Supporting Information 1: Tables S14 and S16. The figures present results from models with a linear specification for the depression and mood disorders polygenic scores and a non‐linear specification (linear and squared term) for the bipolar disorder polygenic score.

We next examined equivalent income as an economic outcome following education and labour market engagement. Ordered probit models showed negative monotonic associations of DPGS and MDPGS with equivalent income across genders (*p* < 0.005; Figure 3, Supporting Information 1: Table S14). No significant PGS–gender interaction terms were observed, suggesting consistent effects across genders. Individuals in the highest DPGS and MDPGS deciles were 4.4 pp (*p* < 0.001; 99.5% CI: 0.9–7.9 pp) and 4.9 pp (*p* < 0.001; 99.5% CI: 1.3–8.3 pp), respectively, less likely to belong to the top income tertile than those in the lowest decile (Supporting Information 1: Table S15). BDPGS showed an inverse U‐shaped relationship with the highest equivalent income tertile (Figure 3, Supporting Information 1: Table S16), but these associations lacked statistical significance.

### 3.5. Equivalent Income: Genetic Associations Beyond Education‐Related Pathways

The ordered probit models stratified by educational categories (Supporting Information 1: Tables S17–S20) provided additional insights by showing associations between PGSs and equivalent income that extend beyond those attributable to educational level. These models revealed negative monotonic associations of BDPGS and MDPGS with equivalent income within the secondary education category, particularly in males (*p* < 0.001). For DPGS, no statistically significant association with equivalent income within the educational categories was found.

### 3.6. Economic Satisfaction and Total Genetic Associations

**Figure 4:**
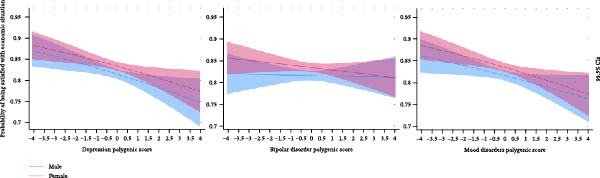
Polygenic scores for depression, bipolar disorder and mood disorders and predicted probabilities of satisfaction with their economic situation in the pooled 1992–2017 sample (*N* = 20,029) with 99.5% CI. Note: The figure presents predicted probabilities from the probit regression models presented in Supporting Information 1: Table S21.

Finally, we studied economic satisfaction as a subjective measure of economic well‐being. Probit models revealed negative monotonic associations for DPGS and MDPGS with economic satisfaction across genders (*p* < 0.005; Figure 4, Supporting Information 1: Table S21). In the highest deciles of DPGS and MDPGS, individuals were 5.3 pp (*p* < 0.001; 99.5% CI: 2.0–8.6 pp) and 5.1 pp (*p* < 0.001; 99.5% CI: 1.8–8.4 pp) less likely to report satisfaction, respectively, than those in the lowest decile (Supporting Information 1: Table S22). BDPGS was not significantly associated with economic satisfaction. No statistically or economically significant non‐linear associations were found between any PGS and economic satisfaction (Supporting Information 1: Table S23).

### 3.7. Economic Satisfaction: Genetic Associations Beyond Income‐Related Pathways

We examined whether the associations between PGSs and economic satisfaction varied by equivalent income level, recognising the previously observed relationships between PGSs and income. Among individuals in the lowest equivalent income tertile (studied using probit models, Supporting Information 1: Table S24), a negative association between DPGS and economic satisfaction persisted (*p* = 0.004). This linear association showed no significant gender differences, as indicated by the non‐significant interaction term between gender and DPGS. However, in separate analyses for males and females, which allows for observing gender‐specific effects of all covariates, the negative association was statistically significant only in males (*p* = 0.003) but not in females, pointing to potential gender‐specific gene–environment correlations or interactions.

The associations between DPGS and economic satisfaction, beyond those attributable to equivalent income level, may operate through education‐related pathways. Descriptive statistics in Supporting Information 1: Figure S5 reveal lower economic satisfaction in the lowest equivalent income tertile among individuals with higher education, suggesting that low returns to education are linked to reduced economic satisfaction. In contrast, no statistically significant associations with economic satisfaction were observed for individuals in the medium or highest equivalent income tertiles (Supporting Information 1: Tables S25–S26).

BDPGS and MDPGS showed no statistically significant associations with economic satisfaction within any equivalent income tertile (Supporting Information 1: Tables S24–S26). These findings indicate that the relationship between MDPGS and economic satisfaction primarily operates through income‐related pathways, whereas no evidence of an association between BDPGS and economic satisfaction was observed.

### 3.8. Sensitivity Analysis With Additional Stratification

To assess the robustness of the results, we performed additional analyses excluding individuals with any diagnosed lifetime mental, behavioural or neurodevelopmental disorders. Diagnostic information was obtained from the Finnish Institute for Health and Welfare’s Care Register for Health Care, based on ICD‐10 F‐category codes and their equivalents in earlier ICD versions. The exclusion thus covered all individuals who had received clinical treatment for any psychiatric disorder up to the study year. Among this subpopulation without recorded lifetime mental disorders, the negative association of DPGS with educational attainment and the positive association of BDPGS persisted, while MDPGS continued to show no significant relationship (Supporting Information 1: Table S27). These results indicate that the observed associations were not primarily driven by diagnosed psychiatric conditions, suggesting that they reflect broader genetic pathways in the general population rather than being mediated by manifest mental disorders.

In addition, to account for potential residual population substructure within the Finnish sample, we repeated the main analyses controlling for the first 10 genetic principal components (PC1–10). The estimates remained consistent with the baseline models using PC1–3, and the additional components contributed only marginal improvements to model fit (Supporting Information 1: Tables S28–S30). This confirms that adjusting for PC1–3 is sufficient in the context of Finland’s relatively homogeneous population structure and that the findings are robust to alternative population stratification controls.

## 4. Discussion

Our study, examining merged genetic, registry and survey data from six Finnish cohorts (1992–2017, *N* = 20,121), reveals complex relationships between polygenic risks for mood disorders and economic outcomes in the general population. The findings demonstrate distinct pathways through which genetic predispositions to depression and bipolar disorder relate to educational attainment, employment status, occupational choice, income and economic satisfaction.

### 4.1. Educational Attainment and Genetic Associations

Educational attainment, a critical mediator of economic success, showed opposing associations with depression and bipolar disorder polygenic scores. We found a significant negative association with DPGS (*p* < 0.001) and a suggested positive association with BDPGS (*p* = 0.02), consistent with previous research indicating negative genetic correlations between educational outcomes and depression [23, 42–44] and positive genetic correlations with BD [18, 23, 43, 45]. These findings also align with a recent study on the positive relationship between genetic risk for BD and actual educational attainment [46]. The magnitude of these associations is substantial, with 4–6 pp differences between higher and lowest polygenic score deciles, relative to the sample’s 38% baseline likelihood of higher educational attainment. Notably, the mood disorders polygenic score, which captures both depression and bipolar disorder risks, showed no significant association with educational attainment, likely due to the offsetting effects of its components. The genetic underpinnings of specific mental disorders, rather than generalised measures, appear to offer more precise insights into variations in educational attainment and the resulting economic disparities.

### 4.2. Labour Market Status and Genetic Associations

Employment status demonstrated significant negative associations with all three PGS (DPGS, BDPGS and MDPGS) across genders (*p* < 0.005). The 6–7 pp differences (*p* < 0.001) in employment probability between highest and lowest PGS deciles are substantial, considering the sample’s 25% baseline non‐employment rate. These findings extend previous research, which reported positive genetic correlations between bipolar disorder and work inability (*rg* = 0.23), negative genetic correlations between depression and socio‐economic status (*rg* = −0.42) and positive associations of depression genetic risks with unemployment experience in middle adulthood [18, 28, 47].

Moreover, the finding that BDPGS and MDPGS are negatively associated with employment through pathways extending beyond educational level suggests that cognitive and non‐cognitive skills [31], including those reflecting the quality of education or other traits captured by BDPGS, may hinder labour market participation despite the positive association of BDPGS with educational attainment. This insight helps reconcile the mixed findings from previous research—discussed in the Introduction—on the genetic correlations of BD with indicators of educational attainment [23], cognitive functioning [27], intelligence [18] and economic outcomes [18, 23, 28].

The opposite‐directional associations of DPGS and BDPGS with educational level are further echoed in selection into knowledge work. Females in the highest DPGS decile were 6 pps less likely to engage in knowledge work (*p* < 0.001)—a notable difference considering the baseline rate of 17%. This finding broadly aligns with previously documented negative genetic correlations between depression and socio‐economic status [28]. Conversely, higher BDPGS was associated with increased likelihood of knowledge work (3 pp increase in highest vs lowest BDPGS decile; *p* = 0.004) but decreased likelihood of physical work in both genders and office work in females (*p* < 0.001), consistent with past research showing positive genetic correlations of BD with educational outcomes and socio‐economic status [18, 23, 28, 48] and positive association between BD polygenic score and creative work [49].

Males with higher DPGS and MDPGS showed reduced likelihood of self‐employment (*p* < 0.005), with a 4 pp difference between extreme deciles relative to the 12% baseline male self‐employment rate, including through pathways that extend beyond educational level. The mechanisms behind that novel finding warrant further study. The absence of evidence for a similar association in females may reflect their overall lower self‐employment rate of 7%.

### 4.3. Income, Economic Satisfaction and Genetic Associations

Income analysis revealed that both DPGS and MDPGS showed negative associations with equivalent income (*p* < 0.005), with individuals in the highest PGS deciles experiencing a 4–5 pp reduction in the likelihood of belonging to the top income tertile compared to those in the lowest deciles. This represents a considerable difference, given the baseline probability of ~33% for each tertile. BDPGS showed no significant net association with equivalent income, as its suggested positive associations with education were counterbalanced by negative associations with equivalent income that extended beyond educational attainment, indicating that the traits proxied by BDPGS may hinder the ability to generate returns to education. These findings align with previous research showing income to have a moderate negative genetic correlation with depression (*rg* = −0.24) and a weak positive genetic correlation with BDPGS (*rg* = 0.07) [23].

Economic satisfaction closely mirrored income patterns, with those in the highest DPGS and MDPGS deciles showing a 5 pp lower likelihood of economic satisfaction (*p* < 0.001), and BDPGS revealing no significant association with economic satisfaction. The negative association between DPGS and economic satisfaction remained significant among individuals in the lowest equivalent income tertile (*p* = 0.004), indicating additional pathways, potentially related to education, that contribute to lower economic satisfaction in those with elevated DPGS. These results align with past research showing negative genetic correlation of DPGS with subjective well‐being (*rg* = −0.64) [21] and mixed evidence for BDPGS, which demonstrates negative correlation with subjective well‐being (*rg* = −0.25) but positive correlation with satisfaction with financial situation (*rg* = 0.10) and lower odds of experiencing major financial troubles [18, 50].

### 4.4. Bidirectional Links Between Socio‐economic Status and Mental Health

Recent evidence highlights the complex, bidirectional interplay between socio‐economic status and mental disorders. Socio‐economic adversity can increase vulnerability to mental disorders through mechanisms such as chronic stress, health inequalities and limited access to care. Conversely, psychiatric traits may contribute to assortative mating, participation bias, downward social mobility and income loss—processes that reinforce cumulative disadvantage and reveal heritable components of both mental health and socio‐economic outcomes [51–54]. Furthermore, these studies indicate that genetic and environmental pathways shaping socio‐economic and mental health trajectories intersect and may influence the population‐level distributions of PGS. Together, these insights suggest that the associations between genetic proxies for mood disorders and economic outcomes may partly reflect shared mechanisms linking psychiatric vulnerability with socio‐economic stratification, underscoring the need for further research into mediating and moderating factors.

### 4.5. Study Limitations and Future Directions

PGSs have inherent limitations as genetic proxies. They explain only a small portion of mood disorder expression and cannot account for environmental influences, gene–environment interactions, gene–environment correlations, pleiotropy or genetic variations beyond SNPs [55]. While PGSs cannot perfectly distinguish between direct genetic effects, genetic nurture and childhood environment, they help identify disorder risk factors that lie outside individual control. Environmental factors remain the primary drivers of economic outcomes, and the causal pathways between genetic proxies and economic outcomes remain latent [56]. Although pleiotropy implies that genetic markers included in a PGS contribute to and correlate with outcomes beyond a specific mood disorder, this does not undermine the objective of our study, which is to examine the associations of DPGS, BDPGS and MDPGS with measures of economic well‐being.

Despite our cohorts’ regional representativeness, some selection bias may arise from voluntary participation in surveys and genetic sampling. Additionally, genetic proxies derived from UK samples may not perfectly capture the Finnish context. Future research should explore neurophysiological mechanisms related to genetic proxies for mood disorders and their interactions with environmental factors affecting economic well‐being.

## 5. Conclusion

Our findings demonstrate that genetic predispositions to mood disorders relate to economic well‐being through multiple, sometimes opposing pathways within the general population. While the observed associations were considerable, they point to meaningful patterns that can inform efforts to create supportive environments mitigating potential disadvantages. Rather than implying deterministic effects, these results highlight how common, continuously distributed genetic liabilities to mood disorders may interact with social and institutional contexts. Understanding these dynamics can help to promote inclusion and resilience while helping to reduce stigma surrounding mental health‐related differences.

## Author Contributions

Aaro Hazak and Tiina Paunio obtained funding for the study and developed the study conception and design. Katri Kantojärvi, Veikko Salomaa, Seppo Koskinen, Markus Perola and Tuija Jääskeläinen prepared, collected and provided the material and data. Aaro Hazak performed the data analysis. Merike Kukk provided methodological guidance. Aaro Hazak and Johanna Liuhanen wrote the manuscript.

## Funding

This project has received funding from the European Union’s Horizon 2020 Research and Innovation Programme (Grant 952574), the Research Council of Finland (Grants 336234 and 357643) and the Helsinki University Hospital (Grant TYH2019315). Open access publishing facilitated by Helsingin yliopisto, as part of the Wiley ‐ FinELib agreement.

## Disclosure

All authors commented on previous versions of the manuscript and read and approved the final manuscript.

## Ethics Statement

Approval for this study was granted by the Finnish Institute for Health and Welfare, and the FR and FH study protocols were approved by the Finnish Institute for Health and Welfare and/or the Coordinating Ethical Committee of the Helsinki and Uusimaa Hospital District (Approval Numbers 38/96, 558/E3/2001, 229/E0/06, 162/13/03/00/2011 and 37/13/03/00/2016). All participants provided written informed consent, and the study adhered to the Declaration of Helsinki.

## Conflicts of Interest

Tiina Paunio: Idorsia Pharmaceuticals and Biogen (unrelated to the present work). The rest of the authors declare no conflicts of interest.

## Supporting Information

Additional supporting information can be found online in the Supporting Information section.

## Supporting information


**Supporting Information 1** Supporting information Tables and Figures.

**Supporting Information 2** STROBE Statement—checklist of items that should be included in reports of observational studies.

## Data Availability

The FR and FH data that support the findings of this study are available from the Finnish Institute for Health and Welfare subject to permission.
