# Supplementary material for: Polygenic Risks for Mood Disorders and Economic Well‐being: Study of Finnish Cohorts
Source: Depress Anxiety. 2026 Feb 23;2026:1008569. doi: 10.1155/da/1008569 (PMC12927894; doi:10.1155/da/1008569)
Supplement: Supplementary file 1 — Supporting Information 1 Supporting information Tables and Figures. [file DA-2026-1008569-s002.docx]

**Polygenic risks for mood disorders and economic wellbeing: Study of Finnish cohorts**

**Supporting Information 1: Supporting Information Tables and Figures**

**Supplementary Figure S1** Histograms of Polygenic Scores for Depression, Bipolar Disorder and Mood Disorders by genders in the pooled 1992-2017 sample

**Supplementary Table S1** Pairwise correlation coefficients and their statistical significance

|  | DPGS | BDPGS | MDPGS | Educational level | Labour market status:  Non-employed | Labour market status:  Self-employed | Labour market status:  Physical work | Labour market status:  Office work | Labour market status:  Knowledge work | Equivalent income tertiles | Economic satisfaction | Gender | Age | Birth cohort | Year | PC1 | PC2 |
| --- | --- | --- | --- | --- | --- | --- | --- | --- | --- | --- | --- | --- | --- | --- | --- | --- | --- |
|  |  |  |  |  |  |  |  |  |  |  |  |  |  |  |  |  |  |
| BDPGS | 0.274 |  |  |  |  |  |  |  |  |  |  |  |  |  |  |  |  |
|  | 0.000 |  |  |  |  |  |  |  |  |  |  |  |  |  |  |  |  |
|  |  |  |  |  |  |  |  |  |  |  |  |  |  |  |  |  |  |
| MDPGS | 0.711 | 0.554 |  |  |  |  |  |  |  |  |  |  |  |  |  |  |  |
|  | 0.000 | 0.000 |  |  |  |  |  |  |  |  |  |  |  |  |  |  |  |
|  |  |  |  |  |  |  |  |  |  |  |  |  |  |  |  |  |  |
| Educational level | -0.038 | 0.014 | -0.013 |  |  |  |  |  |  |  |  |  |  |  |  |  |  |
|  | 0.000 | 0.053 | 0.072 |  |  |  |  |  |  |  |  |  |  |  |  |  |  |
|  |  |  |  |  |  |  |  |  |  |  |  |  |  |  |  |  |  |
| Labour market status: | 0.050 | 0.036 | 0.049 | -0.206 |  |  |  |  |  |  |  |  |  |  |  |  |  |
| Non-employed | 0.000 | 0.000 | 0.000 | 0.000 |  |  |  |  |  |  |  |  |  |  |  |  |  |
|  |  |  |  |  |  |  |  |  |  |  |  |  |  |  |  |  |  |
| Labour market status: | -0.020 | -0.003 | -0.017 | -0.059 | -0.175 |  |  |  |  |  |  |  |  |  |  |  |  |
| Self-employed | 0.005 | 0.716 | 0.017 | 0.000 | 0.000 |  |  |  |  |  |  |  |  |  |  |  |  |
|  |  |  |  |  |  |  |  |  |  |  |  |  |  |  |  |  |  |
| Labour market status: | 0.000 | -0.035 | -0.014 | -0.266 | -0.288 | -0.158 |  |  |  |  |  |  |  |  |  |  |  |
| Physical work | 0.999 | 0.000 | 0.045 | 0.000 | 0.000 | 0.000 |  |  |  |  |  |  |  |  |  |  |  |
|  |  |  |  |  |  |  |  |  |  |  |  |  |  |  |  |  |  |
| Labour market status: | -0.001 | -0.017 | -0.010 | 0.143 | -0.346 | -0.191 | -0.314 |  |  |  |  |  |  |  |  |  |  |
| Office work | 0.917 | 0.014 | 0.152 | 0.000 | 0.000 | 0.000 | 0.000 |  |  |  |  |  |  |  |  |  |  |
|  |  |  |  |  |  |  |  |  |  |  |  |  |  |  |  |  |  |
| Labour market status: | -0.039 | 0.021 | -0.012 | 0.400 | -0.255 | -0.140 | -0.231 | -0.278 |  |  |  |  |  |  |  |  |  |
| Knowledge work | 0.000 | 0.003 | 0.082 | 0.000 | 0.000 | 0.000 | 0.000 | 0.000 |  |  |  |  |  |  |  |  |  |
|  |  |  |  |  |  |  |  |  |  |  |  |  |  |  |  |  |  |
| Equivalent income | -0.040 | -0.016 | -0.031 | 0.293 | -0.361 | -0.026 | -0.035 | 0.141 | 0.327 |  |  |  |  |  |  |  |  |
| tertiles | 0.000 | 0.028 | 0.000 | 0.000 | 0.000 | 0.000 | 0.000 | 0.000 | 0.000 |  |  |  |  |  |  |  |  |
|  |  |  |  |  |  |  |  |  |  |  |  |  |  |  |  |  |  |
| Economic satisfaction | -0.034 | -0.007 | -0.032 | 0.050 | -0.159 | 0.020 | 0.016 | 0.054 | 0.103 | 0.270 |  |  |  |  |  |  |  |
|  | 0.000 | 0.349 | 0.000 | 0.000 | 0.000 | 0.004 | 0.026 | 0.000 | 0.000 | 0.000 |  |  |  |  |  |  |  |
|  |  |  |  |  |  |  |  |  |  |  |  |  |  |  |  |  |  |
| Gender | 0.012 | 0.013 | 0.012 | 0.083 | -0.036 | -0.085 | -0.145 | 0.227 | -0.011 | -0.043 | 0.021 |  |  |  |  |  |  |
| (Male = 0, Female = 1) | 0.099 | 0.076 | 0.085 | 0.000 | 0.000 | 0.000 | 0.000 | 0.000 | 0.106 | 0.000 | 0.003 |  |  |  |  |  |  |
|  |  |  |  |  |  |  |  |  |  |  |  |  |  |  |  |  |  |
| Age | 0.009 | 0.004 | 0.015 | -0.263 | 0.212 | 0.031 | -0.071 | -0.097 | -0.055 | 0.044 | 0.098 | -0.035 |  |  |  |  |  |
|  | 0.194 | 0.585 | 0.031 | 0.000 | 0.000 | 0.000 | 0.000 | 0.000 | 0.000 | 0.000 | 0.000 | 0.000 |  |  |  |  |  |
|  |  |  |  |  |  |  |  |  |  |  |  |  |  |  |  |  |  |
| Birth cohort | -0.012 | -0.004 | -0.013 | 0.341 | -0.199 | -0.021 | 0.032 | 0.085 | 0.092 | -0.029 | -0.064 | 0.023 | -0.795 |  |  |  |  |
| (dummies, 1920ies to 2000s) | 0.099 | 0.537 | 0.069 | 0.000 | 0.000 | 0.003 | 0.000 | 0.000 | 0.000 | 0.000 | 0.000 | 0.001 | 0.000 |  |  |  |  |
|  |  |  |  |  |  |  |  |  |  |  |  |  |  |  |  |  |  |
| Year | -0.006 | 0.001 | 0.001 | 0.221 | -0.052 | 0.007 | -0.046 | 0.014 | 0.081 | 0.021 | 0.028 | -0.011 | 0.038 | 0.540 |  |  |  |
| (study year, 1992 to 2017) | 0.430 | 0.940 | 0.923 | 0.000 | 0.000 | 0.326 | 0.000 | 0.044 | 0.000 | 0.004 | 0.000 | 0.122 | 0.000 | 0.000 |  |  |  |
|  |  |  |  |  |  |  |  |  |  |  |  |  |  |  |  |  |  |
| PC1 | -0.108 | -0.105 | -0.055 | 0.056 | -0.061 | -0.014 | -0.015 | 0.009 | 0.079 | 0.080 | -0.015 | -0.002 | -0.008 | 0.032 | 0.046 |  |  |
|  | 0.000 | 0.000 | 0.000 | 0.000 | 0.000 | 0.055 | 0.030 | 0.192 | 0.000 | 0.000 | 0.038 | 0.835 | 0.276 | 0.000 | 0.000 |  |  |
|  |  |  |  |  |  |  |  |  |  |  |  |  |  |  |  |  |  |
| PC2 | 0.008 | 0.029 | 0.010 | -0.045 | 0.000 | -0.004 | 0.013 | -0.001 | -0.009 | -0.014 | -0.017 | 0.005 | 0.007 | -0.063 | -0.102 | 0.044 |  |
|  | 0.290 | 0.000 | 0.141 | 0.000 | 0.958 | 0.531 | 0.072 | 0.931 | 0.186 | 0.049 | 0.015 | 0.446 | 0.346 | 0.000 | 0.000 | 0.000 |  |
|  |  |  |  |  |  |  |  |  |  |  |  |  |  |  |  |  |  |
| PC3 | 0.041 | 0.018 | 0.039 | 0.010 | 0.009 | -0.010 | -0.004 | 0.007 | -0.008 | -0.029 | 0.012 | 0.010 | -0.027 | 0.039 | 0.033 | 0.001 | 0.035 |
|  | 0.000 | 0.011 | 0.000 | 0.173 | 0.199 | 0.177 | 0.610 | 0.325 | 0.233 | 0.000 | 0.090 | 0.171 | 0.000 | 0.000 | 0.000 | 0.935 | 0.000 |

*Note: DPGS denotes Depression Polygenic Score [21], BDPGS Bipolar Disorder Polygenic Score [18] and MDPGS Mood Disorders Polygenic Score [22].*

**Supplementary Figure S2** Labour market status by educational categories and genders in the pooled 1992-2017 sample

**Supplementary Figure S3** Equivalent income by educational categories and genders in the pooled 1992-2017 sample

**Supplementary Figure S4** Equivalent income among labour market status categories by genders in the pooled 1992-2017 sample

**Supplementary Figure S5** Economic satisfaction by genders in the pooled 1992-2017 sample, by education, labour market status and equivalent income categories

Education

**Supplementary Table S2** Coefficient estimates from ordered probit models of education in the pooled 1992-2017 sample

| **Sample** | **All** | **All** | **Males** | **Females** | **All** | **All** | **Males** | **Females** | **All** | **All** | **Males** | **Females** |
| --- | --- | --- | --- | --- | --- | --- | --- | --- | --- | --- | --- | --- |
| **PGS** | **DPGS** | **DPGS** | **DPGS** | **DPGS** | **BDPGS** | **BDPGS** | **BDPGS** | **BDPGS** | **MDPGS** | **MDPGS** | **MDPGS** | **MDPGS** |
| PGS specification | linear | linear + squared | linear | linear | linear | linear + squared | linear | linear | linear | linear + squared | linear | linear |
| N | 20,121 | 20,121 | 9,248 | 10,873 | 20,121 | 20,121 | 9,248 | 10,873 | 20,121 | 20,121 | 9,248 | 10,873 |
| Model | oprobit | oprobit | oprobit | oprobit | oprobit | oprobit | oprobit | oprobit | oprobit | oprobit | oprobit | oprobit |
| **Dependent variable: Education** | | | | | | | | | | | | |
| Explanatory variables: | | | | | | | | | | | | |
| PGS | -0.044*** | -0.039*** | -0.042*** | -0.036** | 0.027* | 0.023** | 0.028* | 0.019 | -0.003 | -0.010 | -0.004 | -0.017 |
|  | (0.012) | (0.008) | (0.012) | (0.011) | (0.012) | (0.008) | (0.012) | (0.011) | (0.012) | (0.008) | (0.012) | (0.011) |
| PGS squared |  | -0.004 |  |  |  | -0.011 |  |  |  | -0.007 |  |  |
|  |  | (0.006) |  |  |  | (0.006) |  |  |  | (0.006) |  |  |
| Female | 0.196*** | 0.196*** |  |  | 0.195*** | 0.195*** |  |  | 0.196*** | 0.196*** |  |  |
|  | (0.016) | (0.016) |  |  | (0.016) | (0.016) |  |  | (0.016) | (0.016) |  |  |
| PGS # Female | 0.010 |  |  |  | -0.006 |  |  |  | -0.012 |  |  |  |
|  | (0.016) |  |  |  | (0.016) |  |  |  | (0.016) |  |  |  |
| Birth cohort 1920-1930ies | -0.976*** | -0.976*** | -0.808*** | -1.122*** | -0.976*** | -0.976*** | -0.808*** | -1.122*** | -0.976*** | -0.976*** | -0.808*** | -1.123*** |
|  | (0.035) | (0.035) | (0.052) | (0.048) | (0.035) | (0.035) | (0.052) | (0.048) | (0.035) | (0.035) | (0.052) | (0.048) |
| Birth cohort 1940ies | -0.441*** | -0.441*** | -0.353*** | -0.516*** | -0.440*** | -0.441*** | -0.351*** | -0.516*** | -0.440*** | -0.440*** | -0.352*** | -0.515*** |
|  | (0.027) | (0.027) | (0.039) | (0.036) | (0.027) | (0.027) | (0.039) | (0.036) | (0.027) | (0.027) | (0.039) | (0.036) |
| Birth cohort 1950ies | Reference | Reference | Reference | Reference | Reference | Reference | Reference | Reference | Reference | Reference | Reference | Reference |
| Birth cohort 1960ies | 0.263*** | 0.263*** | 0.168*** | 0.347*** | 0.262*** | 0.262*** | 0.168*** | 0.346*** | 0.263*** | 0.262*** | 0.168*** | 0.347*** |
|  | (0.022) | (0.022) | (0.032) | (0.030) | (0.022) | (0.022) | (0.032) | (0.030) | (0.022) | (0.022) | (0.032) | (0.030) |
| Birth cohort 1970ies | 0.398*** | 0.398*** | 0.314*** | 0.470*** | 0.399*** | 0.399*** | 0.316*** | 0.469*** | 0.399*** | 0.398*** | 0.315*** | 0.470*** |
|  | (0.024) | (0.024) | (0.035) | (0.034) | (0.024) | (0.024) | (0.035) | (0.034) | (0.024) | (0.024) | (0.035) | (0.034) |
| Birth cohort 1980-1990ies | 0.374*** | 0.374*** | 0.281*** | 0.453*** | 0.376*** | 0.375*** | 0.285*** | 0.454*** | 0.375*** | 0.375*** | 0.283*** | 0.453*** |
|  | (0.029) | (0.030) | (0.042) | (0.041) | (0.030) | (0.030) | (0.043) | (0.041) | (0.029) | (0.029) | (0.042) | (0.041) |
| PC1 | 8.526*** | 8.550*** | 10.970*** | 6.304*** | 9.456*** | 9.531*** | 12.055*** | 7.085*** | 9.035*** | 9.054*** | 11.588*** | 6.704*** |
|  | (1.149) | (1.150) | (1.661) | (1.596) | (1.150) | (1.150) | (1.662) | (1.596) | (1.145) | (1.145) | (1.656) | (1.590) |
| PC2 | -4.134*** | -4.137*** | -2.620 | -5.699*** | -4.305*** | -4.252*** | -2.833 | -5.817*** | -4.183*** | -4.184*** | -2.684 | -5.726*** |
|  | (1.164) | (1.164) | (1.620) | (1.657) | (1.167) | (1.165) | (1.631) | (1.664) | (1.163) | (1.163) | (1.622) | (1.657) |
| PC3 | 0.147 | 0.162 | -0.324 | 0.686 | -0.116 | -0.119 | -0.585 | 0.414 | -0.011 | -0.001 | -0.525 | 0.565 |
|  | (1.098) | (1.099) | (1.567) | (1.544) | (1.099) | (1.098) | (1.567) | (1.546) | (1.098) | (1.098) | (1.568) | (1.544) |
| Cut: Primary/Secondary education | -0.824*** | -0.828*** | -0.843*** | -1.009*** | -0.825*** | -0.836*** | -0.842*** | -1.008*** | -0.824*** | -0.831*** | -0.842*** | -1.008*** |
|  | (0.019) | (0.020) | (0.024) | (0.024) | (0.019) | (0.020) | (0.024) | (0.025) | (0.019) | (0.020) | (0.024) | (0.024) |
| Cut: Secondary/Higher education | 0.445*** | 0.442*** | 0.443*** | 0.255*** | 0.444*** | 0.433*** | 0.442*** | 0.255*** | 0.445*** | 0.438*** | 0.442*** | 0.255*** |
|  | (0.018) | (0.019) | (0.024) | (0.023) | (0.018) | (0.019) | (0.024) | (0.023) | (0.018) | (0.019) | (0.024) | (0.023) |
|  |  |  |  |  |  |  |  |  |  |  |  |  |
| Pseudo-R^2^ | 0.071 | 0.071 | 0.045 | 0.091 | 0.070 | 0.070 | 0.045 | 0.091 | 0.070 | 0.070 | 0.044 | 0.091 |
| p(χ^2^) | *** | *** | *** | *** | *** | *** | *** | *** | *** | *** | *** | *** |

** P < 0.05, ** P < 0.005, *** P < 0.001; robust SE in parentheses; DPGS denotes Depression Polygenic Score [21], BDPGS Bipolar Disorder Polygenic Score [18] and MDPGS Mood Disorders Polygenic Score [22].*

**Supplementary Table S3** Differences in predicted probabilities of having a specific level of education in the DPGS and BDPGS top three deciles compared to the lowest decile in the pooled 1992-2017 sample

| **Sample** | **All** | **Males** | **Females** | **All** | **Males** | **Females** |
| --- | --- | --- | --- | --- | --- | --- |
| **PGS** | **DPGS** | **DPGS** | **DPGS** | **BDPGS** | **BDPGS** | **BDPGS** |
| Total N in the model | 20,121 | 9,248 | 10,873 | 20,121 | 9,248 | 10,873 |
| **Reference: 1^st^ decile PGS** |  |  |  |  |  |  |
| Average marginal effects: |  |  |  |  |  |  |
| **8^th^ decile PGS** |  |  |  |  |  |  |
| Primary education | 0.023* | 0.044** | 0.007 | -0.020* | -0.037* | -0.009 |
|  | (0.009) | (0.014) | (0.011) | (0.009) | (0.015) | (0.012) |
| Secondary education | 0.010* | 0.014** | 0.004 | -0.008* | -0.009* | -0.004 |
|  | (0.004) | (0.005) | (0.006) | (0.003) | (0.004) | (0.006) |
| Higher education | -0.033* | -0.058** | -0.011 | 0.028* | 0.045* | 0.014 |
|  | (0.013) | (0.018) | (0.018) | (0.013) | (0.018) | (0.017) |
| **9^th^ decile PGS** |  |  |  |  |  |  |
| Primary education | 0.023* | 0.031* | 0.015 | -0.027** | -0.034* | -0.020 |
|  | (0.009) | (0.014) | (0.012) | (0.009) | (0.015) | (0.011) |
| Secondary education | 0.010* | 0.011* | 0.008 | -0.010** | -0.008* | -0.010 |
|  | (0.004) | (0.005) | (0.006) | (0.003) | (0.004) | (0.006) |
| Higher education | -0.033* | -0.042* | -0.023 | 0.036** | 0.042* | 0.030 |
|  | (0.013) | (0.019) | (0.018) | (0.013) | (0.018) | (0.017) |
| **10^th^ decile PGS** |  |  |  |  |  |  |
| Primary education | 0.045*** | 0.068*** | 0.027* | -0.012 | -0.015 | -0.010 |
|  | (0.009) | (0.015) | (0.012) | (0.009) | (0.015) | (0.012) |
| Secondary education | 0.017*** | 0.018*** | 0.013* | -0.004 | -0.003 | -0.005 |
|  | (0.004) | (0.004) | (0.006) | (0.003) | (0.003) | (0.006) |
| Higher education | -0.062*** | -0.086*** | -0.040* | 0.017 | 0.018 | 0.015 |
|  | (0.013) | (0.018) | (0.017) | (0.013) | (0.018) | (0.017) |

** P < 0.05, ** P < 0.005, *** P < 0.001; robust SE in parentheses; polygenic scores (PGS) for depression (DPGS) [21] and bipolar disorder (BDPGS) [18]; table presenting average marginal effects from ordered probit models of education with deciles of DPGS or BDPGS as main explanatory variable and controls for gender, birth cohort dummies and PC1-3.*

**Supplementary Table S4** Coefficient estimates from probit models of belonging to a specific labour market status in the pooled 1992-2017 sample: Depression Polygenic Score (DPGS)

| **Sample** | **All** | **All** | **All** | **All** | **All** | **All** | **All** | **All** | **All** | **All** |
| --- | --- | --- | --- | --- | --- | --- | --- | --- | --- | --- |
| **PGS** | **DPGS** | **DPGS** | **DPGS** | **DPGS** | **DPGS** | **DPGS** | **DPGS** | **DPGS** | **DPGS** | **DPGS** |
| **PGS specification** | **Linear** | **Linear** | **Linear** | **Linear** | **Linear** | **Linear + squared** | **Linear + squared** | **Linear + squared** | **Linear + squared** | **Linear + squared** |
| **Labour market status** | **Non-employed** | **Self-employed** | **Physical**  **work** | **Office**  **work** | **Knowledge work** | **Non-employed** | **Self-employed** | **Physical**  **work** | **Office**  **work** | **Knowledge work** |
| N | 20,121 | 20,121 | 20,121 | 20,121 | 20,121 | 20,121 | 20,121 | 20,121 | 20,121 | 20,121 |
| Model | probit | probit | probit | probit | probit | probit | probit | probit | probit | probit |
| **Dependent variable: belonging to the specific labour market status** | | | | | | | | | | |
| Explanatory variables: | | | | | | | | | | |
| PGS | 0.065*** | -0.056** | 0.011 | -0.018 | -0.029 | 0.070*** | -0.039** | -0.000 | -0.004 | -0.050*** |
|  | (0.015) | (0.017) | (0.014) | (0.016) | (0.016) | (0.010) | (0.013) | (0.010) | (0.010) | (0.011) |
| PGS squared |  |  |  |  |  | 0.007 | -0.012 | 0.004 | 0.010 | -0.020* |
|  |  |  |  |  |  | (0.007) | (0.009) | (0.007) | (0.007) | (0.008) |
| Female | -0.110*** | -0.293*** | -0.421*** | 0.650*** | -0.038 | -0.109*** | -0.295*** | -0.421*** | 0.649*** | -0.036 |
|  | (0.021) | (0.026) | (0.020) | (0.020) | (0.021) | (0.021) | (0.025) | (0.020) | (0.020) | (0.021) |
| PGS # Female | 0.009 | 0.040 | -0.023 | 0.023 | -0.034 |  |  |  |  |  |
|  | (0.021) | (0.025) | (0.021) | (0.020) | (0.021) |  |  |  |  |  |
| Age | -0.252*** | 0.124*** | 0.050*** | 0.069*** | 0.129*** | -0.252*** | 0.124*** | 0.050*** | 0.069*** | 0.129*** |
|  | (0.010) | (0.012) | (0.010) | (0.009) | (0.010) | (0.010) | (0.012) | (0.010) | (0.009) | (0.010) |
| Age squared | 0.003*** | -0.001*** | -0.001*** | -0.001*** | -0.002*** | 0.003*** | -0.001*** | -0.001*** | -0.001*** | -0.002*** |
|  | (0.000) | (0.000) | (0.000) | (0.000) | (0.000) | (0.000) | (0.000) | (0.000) | (0.000) | (0.000) |
| PC1 | -11.943*** | -3.866* | -2.700 | 1.958 | 15.405*** | -11.978*** | -3.788* | -2.735 | 1.924 | 15.483*** |
|  | (1.499) | (1.808) | (1.431) | (1.356) | (1.463) | (1.500) | (1.807) | (1.432) | (1.357) | (1.463) |
| PC2 | 0.825 | -0.668 | 1.685 | -0.670 | -1.706 | 0.824 | -0.648 | 1.691 | -0.664 | -1.733 |
|  | (1.661) | (1.874) | (1.450) | (1.352) | (1.415) | (1.661) | (1.870) | (1.451) | (1.350) | (1.418) |
| PC3 | 3.372* | -2.024 | -0.480 | 0.361 | -2.282 | 3.348* | -1.972 | -0.497 | 0.336 | -2.217 |
|  | (1.468) | (1.804) | (1.443) | (1.305) | (1.377) | (1.468) | (1.805) | (1.444) | (1.305) | (1.377) |
| Year dummies | Yes | Yes | Yes | Yes | Yes | Yes | Yes | Yes | Yes | Yes |
| Birth cohort dummies | Yes | Yes | Yes | Yes | Yes | Yes | Yes | Yes | Yes | Yes |
| Constant | 3.592*** | -3.942*** | -1.245*** | -1.858*** | -3.595*** | 3.586*** | -3.928*** | -1.248*** | -1.867*** | -3.579*** |
|  | (0.238) | (0.310) | (0.236) | (0.224) | (0.248) | (0.238) | (0.310) | (0.236) | (0.224) | (0.248) |
|  |  |  |  |  |  |  |  |  |  |  |
| Pseudo-R^2^ | 0.129 | 0.028 | 0.034 | 0.065 | 0.035 | 0.129 | 0.028 | 0.034 | 0.065 | 0.035 |
| p(χ^2^) | *** | *** | *** | *** | *** | *** | *** | *** | *** | *** |

** P < 0.05, ** P < 0.005, *** P < 0.001; robust SE in parentheses; DPGS denotes Depression Polygenic Score [21]*

**Supplementary Table S5** Coefficient estimates from probit models of belonging to a specific labour market status in the pooled 1992-2017 sample by genders: Depression Polygenic Score (DPGS)

| **Sample** | **Males** | **Males** | **Males** | **Males** | **Males** | **Males** | **Females** | **Females** | **Females** | **Females** | **Females** | **Females** |
| --- | --- | --- | --- | --- | --- | --- | --- | --- | --- | --- | --- | --- |
| **PGS** | **DPGS** | **DPGS** | **DPGS** | **DPGS** | **DPGS** | **DPGS** | **DPGS** | **DPGS** | **DPGS** | **DPGS** | **DPGS** | **DPGS** |
| **PGS specification** | **Linear** | **Linear** | **Linear** | **Linear** | **Linear** | **Linear + squared** | **Linear** | **Linear** | **Linear** | **Linear** | **Linear** | **Linear + squared** |
| **Labour market status** | **Non-employed** | **Self-employed** | **Physical**  **work** | **Office**  **work** | **Knowledge work** | **Knowledge work** | **Non-employed** | **Self-employed** | **Physical**  **work** | **Office**  **work** | **Knowledge work** | **Knowledge work** |
| N | 9,248 | 9,248 | 9,248 | 9,248 | 9,248 | 9,248 | 10,873 | 10,873 | 10,873 | 10,873 | 10,873 | 10,873 |
| Model | probit | probit | probit | probit | probit | probit | probit | probit | probit | probit | probit | probit |
| **Dependent variable: belonging to the specific labour market status** | | | | | | | | | | | | |
| Explanatory variables: | | | | | | | | | | | | |
| PGS | 0.063*** | -0.057** | 0.010 | -0.014 | -0.028 | -0.029 | 0.076*** | -0.016 | -0.011 | 0.003 | -0.066*** | -0.069*** |
|  | (0.015) | (0.018) | (0.014) | (0.016) | (0.016) | (0.016) | (0.014) | (0.018) | (0.015) | (0.013) | (0.014) | (0.015) |
| PGS squared |  |  |  |  |  | -0.011 |  |  |  |  |  | -0.029* |
|  |  |  |  |  |  | (0.011) |  |  |  |  |  | (0.011) |
| Age | -0.238*** | 0.146*** | 0.047*** | 0.053*** | 0.124*** | 0.124*** | -0.263*** | 0.103*** | 0.050*** | 0.077*** | 0.135*** | 0.135*** |
|  | (0.014) | (0.017) | (0.013) | (0.015) | (0.015) | (0.015) | (0.013) | (0.018) | (0.014) | (0.012) | (0.014) | (0.014) |
| Age squared | 0.003*** | -0.001*** | -0.001*** | -0.001*** | -0.001*** | -0.001*** | 0.003*** | -0.001*** | -0.001*** | -0.001*** | -0.002*** | -0.002*** |
|  | (0.000) | (0.000) | (0.000) | (0.000) | (0.000) | (0.000) | (0.000) | (0.000) | (0.000) | (0.000) | (0.000) | (0.000) |
| PC1 | -14.691*** | -3.154 | -4.480* | 5.073* | 18.194*** | 18.239*** | -9.329*** | -5.087 | -0.561 | -0.010 | 12.788*** | 12.948*** |
|  | (2.150) | (2.436) | (1.973) | (2.162) | (2.135) | (2.137) | (2.086) | (2.719) | (2.099) | (1.746) | (2.013) | (2.012) |
| PC2 | -2.005 | -1.769 | 1.211 | 1.479 | 0.281 | 0.293 | 3.468 | 0.998 | 2.517 | -2.256 | -3.671 | -3.756 |
|  | (2.401) | (2.713) | (1.942) | (2.042) | (2.038) | (2.040) | (2.335) | (2.597) | (2.208) | (1.761) | (1.972) | (1.972) |
| PC3 | 3.746 | -0.482 | -2.742 | -0.440 | -1.447 | -1.396 | 2.949 | -4.121 | 1.782 | 1.083 | -2.945 | -2.857 |
|  | (2.142) | (2.464) | (1.940) | (2.083) | (2.016) | (2.016) | (2.017) | (2.708) | (2.174) | (1.681) | (1.898) | (1.898) |
| Year dummies | Yes | Yes | Yes | Yes | Yes | Yes | Yes | Yes | Yes | Yes | Yes | Yes |
| Birth cohort dummies | Yes | Yes | Yes | Yes | Yes | Yes | Yes | Yes | Yes | Yes | Yes | Yes |
| Constant | 3.328*** | -4.582*** | -0.957** | -1.505*** | -3.623*** | -3.613*** | 3.649*** | -3.596*** | -1.896*** | -1.380*** | -3.668*** | -3.645*** |
|  | (0.351) | (0.422) | (0.328) | (0.363) | (0.362) | (0.362) | (0.327) | (0.455) | (0.345) | (0.284) | (0.342) | (0.342) |
|  |  |  |  |  |  |  |  |  |  |  |  |  |
| Pseudo-R^2^ | 0.130 | 0.021 | 0.024 | 0.020 | 0.032 | 0.032 | 0.130 | 0.015 | 0.011 | 0.024 | 0.040 | 0.041 |
| p(χ^2^) | *** | *** | *** | *** | *** | *** | *** | *** | *** | *** | *** | *** |

** P < 0.05, ** P < 0.005, *** P < 0.001; robust SE in parentheses; DPGS denotes Depression Polygenic Score [21]*

**Supplementary Table S6** Coefficient estimates from probit models of belonging to a specific labour market status in the pooled 1992-2017 sample: Bipolar Disorder Polygenic Score (BDPGS)

| **Sample** | **All** | **All** | **All** | **All** | **All** | **All** | **All** | **All** | **All** | **All** |
| --- | --- | --- | --- | --- | --- | --- | --- | --- | --- | --- |
| **PGS** | **BDPGS** | **BDPGS** | **BDPGS** | **BDPGS** | **BDPGS** | **BDPGS** | **BDPGS** | **BDPGS** | **BDPGS** | **BDPGS** |
| **PGS specification** | **Linear** | **Linear** | **Linear** | **Linear** | **Linear** | **Linear + squared** | **Linear + squared** | **Linear + squared** | **Linear + squared** | **Linear + squared** |
| **Labour market status** | **Non-employed** | **Self-employed** | **Physical**  **work** | **Office**  **work** | **Knowledge work** | **Non-employed** | **Self-employed** | **Physical**  **work** | **Office**  **work** | **Knowledge work** |
| N | 20,121 | 20,121 | 20,121 | 20,121 | 20,121 | 20,121 | 20,121 | 20,121 | 20,121 | 20,121 |
| Model | probit | probit | probit | probit | probit | probit | probit | probit | probit | probit |
| **Dependent variable: belonging to the specific labour market status** | | | | | | | | | | |
| Explanatory variables: | | | | | | | | | | |
| PGS | 0.055*** | -0.016 | -0.062*** | -0.009 | 0.039* | 0.051*** | -0.005 | -0.051*** | -0.029** | 0.045*** |
|  | (0.015) | (0.017) | (0.014) | (0.016) | (0.015) | (0.010) | (0.013) | (0.010) | (0.010) | (0.011) |
| PGS squared |  |  |  |  |  | 0.018* | 0.002 | 0.001 | -0.010 | -0.016* |
|  |  |  |  |  |  | (0.007) | (0.009) | (0.007) | (0.007) | (0.007) |
| Female | -0.109*** | -0.296*** | -0.419*** | 0.649*** | -0.038 | -0.109*** | -0.296*** | -0.420*** | 0.650*** | -0.038 |
|  | (0.021) | (0.026) | (0.020) | (0.020) | (0.021) | (0.021) | (0.025) | (0.020) | (0.020) | (0.021) |
| PGS # Female | -0.009 | 0.023 | 0.023 | -0.030 | 0.012 |  |  |  |  |  |
|  | (0.021) | (0.026) | (0.020) | (0.020) | (0.021) |  |  |  |  |  |
| Age | -0.251*** | 0.124*** | 0.050*** | 0.069*** | 0.129*** | -0.251*** | 0.124*** | 0.050*** | 0.069*** | 0.129*** |
|  | (0.010) | (0.012) | (0.010) | (0.009) | (0.010) | (0.010) | (0.012) | (0.010) | (0.009) | (0.010) |
| Age squared | 0.003*** | -0.001*** | -0.001*** | -0.001*** | -0.002*** | 0.003*** | -0.001*** | -0.001*** | -0.001*** | -0.002*** |
|  | (0.000) | (0.000) | (0.000) | (0.000) | (0.000) | (0.000) | (0.000) | (0.000) | (0.000) | (0.000) |
| PC1 | -12.203*** | -3.364 | -3.492* | 1.636 | 16.763*** | -12.339*** | -3.369 | -3.483* | 1.696 | 16.879*** |
|  | (1.497) | (1.810) | (1.432) | (1.355) | (1.469) | (1.498) | (1.810) | (1.433) | (1.356) | (1.470) |
| PC2 | 0.662 | -0.697 | 1.944 | -0.556 | -2.055 | 0.586 | -0.730 | 1.933 | -0.523 | -1.967 |
|  | (1.658) | (1.849) | (1.466) | (1.352) | (1.415) | (1.655) | (1.836) | (1.465) | (1.357) | (1.416) |
| PC3 | 3.729* | -2.193 | -0.387 | 0.418 | -2.648 | 3.722* | -2.178 | -0.374 | 0.408 | -2.650 |
|  | (1.465) | (1.798) | (1.446) | (1.305) | (1.377) | (1.465) | (1.795) | (1.446) | (1.305) | (1.377) |
| Year dummies | Yes | Yes | Yes | Yes | Yes | Yes | Yes | Yes | Yes | Yes |
| Birth cohort dummies | Yes | Yes | Yes | Yes | Yes | Yes | Yes | Yes | Yes | Yes |
| Constant | 3.572*** | -3.939*** | -1.247*** | -1.852*** | -3.597*** | 3.557*** | -3.941*** | -1.246*** | -1.843*** | -3.582*** |
|  | (0.238) | (0.310) | (0.236) | (0.224) | (0.248) | (0.238) | (0.310) | (0.236) | (0.224) | (0.248) |
|  |  |  |  |  |  |  |  |  |  |  |
| Pseudo-R^2^ | 0.128 | 0.027 | 0.035 | 0.065 | 0.035 | 0.129 | 0.027 | 0.035 | 0.065 | 0.035 |
| p(χ^2^) | *** | *** | *** | *** | *** | *** | *** | *** | *** | *** |

** P < 0.05, ** P < 0.005, *** P < 0.001; robust SE in parentheses; BDPGS denotes Bipolar Disorder Polygenic Score [18]*

**Supplementary Table S7** Coefficient estimates from probit models of belonging to a specific labour market status in the pooled 1992-2017 sample by genders: Bipolar Disorder Polygenic Score (BDPGS)

| **Sample** | **Males** | **Males** | **Males** | **Males** | **Males** | **Males** | **Males** | **Females** | **Females** | **Females** | **Females** | **Females** | **Females** | **Females** |
| --- | --- | --- | --- | --- | --- | --- | --- | --- | --- | --- | --- | --- | --- | --- |
| **PGS** | **BDPGS** | **BDPGS** | **BDPGS** | **BDPGS** | **BDPGS** | **BDPGS** | **BDPGS** | **BDPGS** | **BDPGS** | **BDPGS** | **BDPGS** | **BDPGS** | **BDPGS** | **BDPGS** |
| **PGS specification** | **Linear** | **Linear + squared** | **Linear** | **Linear** | **Linear** | **Linear** | **Linear + squared** | **Linear** | **Linear + squared** | **Linear** | **Linear** | **Linear** | **Linear** | **Linear + squared** |
| **Labour market status** | **Non-employed** | **Non-employed** | **Self-employed** | **Physical**  **work** | **Office**  **work** | **Knowledge work** | **Knowledge work** | **Non-employed** | **Non-employed** | **Self-employed** | **Physical**  **work** | **Office**  **work** | **Knowledge work** | **Knowledge work** |
| N | 9,248 | 9,248 | 9,248 | 9,248 | 9,248 | 9,248 | 9,248 | 10,873 | 10,873 | 10,873 | 10,873 | 10,873 | 10,873 | 10,873 |
| Model | probit | probit | probit | probit | probit | probit | probit | probit | probit | probit | probit | probit | probit | probit |
| **Dependent variable: belonging to the specific labour market status** | | | | | | | | | | | | | | |
| Explanatory variables: | | | | | | | | | | | | | | |
| PGS | 0.054*** | 0.054*** | -0.015 | -0.064*** | -0.006 | 0.040* | 0.039* | 0.048** | 0.048*** | 0.007 | -0.037* | -0.041*** | 0.050*** | 0.049*** |
|  | (0.015) | (0.015) | (0.017) | (0.014) | (0.016) | (0.015) | (0.016) | (0.015) | (0.014) | (0.019) | (0.015) | (0.012) | (0.015) | (0.015) |
| PGS squared |  | 0.012 |  |  |  |  | -0.016 |  | 0.023* |  |  |  |  | -0.016 |
|  |  | (0.011) |  |  |  |  | (0.011) |  | (0.010) |  |  |  |  | (0.010) |
| Age | -0.237*** | -0.237*** | 0.145*** | 0.048*** | 0.053*** | 0.124*** | 0.124*** | -0.262*** | -0.262*** | 0.103*** | 0.049*** | 0.076*** | 0.135*** | 0.136*** |
|  | (0.014) | (0.014) | (0.017) | (0.013) | (0.015) | (0.015) | (0.015) | (0.013) | (0.013) | (0.018) | (0.014) | (0.012) | (0.014) | (0.014) |
| Age squared | 0.003*** | 0.003*** | -0.001*** | -0.001*** | -0.001*** | -0.001*** | -0.001*** | 0.003*** | 0.003*** | -0.001*** | -0.001*** | -0.001*** | -0.002*** | -0.002*** |
|  | (0.000) | (0.000) | (0.000) | (0.000) | (0.000) | (0.000) | (0.000) | (0.000) | (0.000) | (0.000) | (0.000) | (0.000) | (0.000) | (0.000) |
| PC1 | -14.848*** | -14.946*** | -2.476 | -5.637** | 5.201* | 19.267*** | 19.365*** | -9.671*** | -9.831*** | -4.780 | -0.943 | -0.640 | 14.423*** | 14.553*** |
|  | (2.145) | (2.144) | (2.433) | (1.974) | (2.161) | (2.143) | (2.143) | (2.085) | (2.088) | (2.727) | (2.100) | (1.746) | (2.022) | (2.024) |
| PC2 | -2.239 | -2.339 | -1.788 | 1.553 | 1.477 | -0.030 | 0.105 | 3.360 | 3.349 | 0.954 | 2.674 | -2.073 | -4.065* | -4.000* |
|  | (2.387) | (2.372) | (2.654) | (1.971) | (2.045) | (2.046) | (2.047) | (2.336) | (2.335) | (2.588) | (2.218) | (1.762) | (1.971) | (1.970) |
| PC3 | 4.058 | 4.061 | -0.705 | -2.627 | -0.513 | -1.616 | -1.655 | 3.338 | 3.317 | -4.241 | 1.846 | 1.229 | -3.488 | -3.475 |
|  | (2.137) | (2.135) | (2.449) | (1.946) | (2.081) | (2.014) | (2.014) | (2.013) | (2.011) | (2.703) | (2.175) | (1.682) | (1.898) | (1.899) |
| Year dummies | Yes | Yes | Yes | Yes | Yes | Yes | Yes | Yes | Yes | Yes | Yes | Yes | Yes | Yes |
| Birth cohort dummies | Yes | Yes | Yes | Yes | Yes | Yes | Yes | Yes | Yes | Yes | Yes | Yes | Yes | Yes |
| Constant | 3.309*** | 3.300*** | -4.574*** | -0.961** | -1.503*** | -3.622*** | -3.607*** | 3.628*** | 3.608*** | -3.599*** | -1.891*** | -1.372*** | -3.674*** | -3.658*** |
|  | (0.350) | (0.351) | (0.422) | (0.329) | (0.363) | (0.362) | (0.362) | (0.327) | (0.327) | (0.455) | (0.345) | (0.285) | (0.342) | (0.342) |
|  |  |  |  |  |  |  |  |  |  |  |  |  |  |  |
| Pseudo-R^2^ | 0.130 | 0.130 | 0.019 | 0.026 | 0.020 | 0.032 | 0.032 | 0.128 | 0.129 | 0.015 | 0.011 | 0.024 | 0.039 | 0.040 |
| p(χ^2^) | *** | *** | *** | *** | *** | *** | *** | *** | *** | *** | *** | *** | *** | *** |

** P < 0.05, ** P < 0.005, *** P < 0.001; robust SE in parentheses; BDPGS denotes Bipolar Disorder Polygenic Score [18]*

**Supplementary Table S8** Coefficient estimates from probit models of belonging to a specific labour market status in the pooled 1992-2017 sample: Mood Disorders Polygenic Score (MDPGS)

| **Sample** | **All** | **All** | **All** | **All** | **All** | **All** | **All** | **All** | **All** | **All** |
| --- | --- | --- | --- | --- | --- | --- | --- | --- | --- | --- |
| **PGS** | **MDPGS** | **MDPGS** | **MDPGS** | **MDPGS** | **MDPGS** | **MDPGS** | **MDPGS** | **MDPGS** | **MDPGS** | **MDPGS** |
| **PGS specification** | **Linear** | **Linear** | **Linear** | **Linear** | **Linear** | **Linear + squared** | **Linear + squared** | **Linear + squared** | **Linear + squared** | **Linear + squared** |
| **Labour market status** | **Non-employed** | **Self-employed** | **Physical**  **work** | **Office**  **work** | **Knowledge work** | **Non-employed** | **Self-employed** | **Physical**  **work** | **Office**  **work** | **Knowledge work** |
| N | 20,121 | 20,121 | 20,121 | 20,121 | 20,121 | 20,121 | 20,121 | 20,121 | 20,121 | 20,121 |
| Model | probit | probit | probit | probit | probit | probit | probit | probit | probit | probit |
| **Dependent variable: belonging to the specific labour market status** | | | | | | | | | | |
| Explanatory variables: | | | | | | | | | | |
| PGS | 0.070*** | -0.054** | -0.024 | -0.004 | -0.002 | 0.072*** | -0.030* | -0.017 | -0.017 | -0.013 |
|  | (0.015) | (0.017) | (0.014) | (0.016) | (0.015) | (0.010) | (0.013) | (0.010) | (0.010) | (0.011) |
| PGS squared |  |  |  |  |  | 0.007 | -0.001 | 0.008 | -0.002 | -0.020* |
|  |  |  |  |  |  | (0.007) | (0.009) | (0.007) | (0.007) | (0.008) |
| Female | -0.109*** | -0.294*** | -0.421*** | 0.649*** | -0.037 | -0.109*** | -0.295*** | -0.421*** | 0.649*** | -0.037 |
|  | (0.021) | (0.026) | (0.020) | (0.020) | (0.021) | (0.021) | (0.025) | (0.020) | (0.020) | (0.021) |
| PGS # Female | 0.005 | 0.054* | 0.012 | -0.021 | -0.019 |  |  |  |  |  |
|  | (0.021) | (0.026) | (0.021) | (0.020) | (0.021) |  |  |  |  |  |
| Age | -0.252*** | 0.124*** | 0.050*** | 0.069*** | 0.128*** | -0.252*** | 0.124*** | 0.050*** | 0.069*** | 0.129*** |
|  | (0.010) | (0.012) | (0.010) | (0.009) | (0.010) | (0.010) | (0.012) | (0.010) | (0.009) | (0.010) |
| Age squared | 0.003*** | -0.001*** | -0.001*** | -0.001*** | -0.002*** | 0.003*** | -0.001*** | -0.001*** | -0.001*** | -0.002*** |
|  | (0.000) | (0.000) | (0.000) | (0.000) | (0.000) | (0.000) | (0.000) | (0.000) | (0.000) | (0.000) |
| PC1 | -12.417*** | -3.561* | -2.857* | 1.922 | 16.024*** | -12.444*** | -3.503 | -2.878* | 1.915 | 16.097*** |
|  | (1.491) | (1.802) | (1.425) | (1.350) | (1.460) | (1.491) | (1.801) | (1.425) | (1.351) | (1.460) |
| PC2 | 0.802 | -0.644 | 1.720 | -0.659 | -1.767 | 0.807 | -0.686 | 1.721 | -0.658 | -1.796 |
|  | (1.658) | (1.867) | (1.454) | (1.350) | (1.413) | (1.658) | (1.860) | (1.454) | (1.352) | (1.415) |
| PC3 | 3.397* | -2.047 | -0.395 | 0.426 | -2.476 | 3.381* | -2.026 | -0.413 | 0.425 | -2.461 |
|  | (1.468) | (1.801) | (1.444) | (1.305) | (1.376) | (1.468) | (1.800) | (1.444) | (1.305) | (1.376) |
| Year dummies | Yes | Yes | Yes | Yes | Yes | Yes | Yes | Yes | Yes | Yes |
| Birth cohort dummies | Yes | Yes | Yes | Yes | Yes | Yes | Yes | Yes | Yes | Yes |
| Constant | 3.591*** | -3.938*** | -1.247*** | -1.858*** | -3.594*** | 3.586*** | -3.937*** | -1.254*** | -1.856*** | -3.580*** |
|  | (0.238) | (0.310) | (0.236) | (0.224) | (0.248) | (0.238) | (0.310) | (0.236) | (0.224) | (0.248) |
|  |  |  |  |  |  |  |  |  |  |  |
| Pseudo-R^2^ | 0.129 | 0.028 | 0.034 | 0.065 | 0.034 | 0.130 | 0.028 | 0.034 | 0.065 | 0.034 |
| p(χ^2^) | *** | *** | *** | *** | *** | *** | *** | *** | *** | *** |

** P < 0.05, ** P < 0.005, *** P < 0.001; robust SE in parentheses; MDPGS denotes Mood Disorders Polygenic Score [22]*

**Supplementary Table S9** Coefficient estimates from probit models of belonging to a specific labour market status in the pooled 1992-2017 sample by genders: Mood Disorders Polygenic Score (MDPGS)

| **Sample** | **Males** | **Males** | **Males** | **Males** | **Males** | **Females** | **Females** | **Females** | **Females** | **Females** |
| --- | --- | --- | --- | --- | --- | --- | --- | --- | --- | --- |
| **PGS** | **MDPGS** | **MDPGS** | **MDPGS** | **MDPGS** | **MDPGS** | **MDPGS** | **MDPGS** | **MDPGS** | **MDPGS** | **MDPGS** |
| **Labour market status** | **Non-employed** | **Self-employed** | **Physical**  **work** | **Office**  **work** | **Knowledge work** | **Non-employed** | **Self-employed** | **Physical**  **work** | **Office**  **work** | **Knowledge work** |
| N | 9,248 | 9,248 | 9,248 | 9,248 | 9,248 | 10,873 | 10,873 | 10,873 | 10,873 | 10,873 |
| Model | probit | probit | probit | probit | probit | probit | probit | probit | probit | probit |
| **Dependent variable: belonging to the specific labour market status** | | | | | | | | | | |
| Explanatory variables: | | | | | | | | | | |
| PGS | 0.069*** | -0.055** | -0.023 | -0.001 | -0.002 | 0.077*** | 0.000 | -0.011 | -0.027* | -0.021 |
|  | (0.015) | (0.017) | (0.014) | (0.016) | (0.015) | (0.015) | (0.019) | (0.015) | (0.013) | (0.014) |
| Age | -0.237*** | 0.145*** | 0.048*** | 0.053*** | 0.124*** | -0.263*** | 0.102*** | 0.050*** | 0.077*** | 0.135*** |
|  | (0.014) | (0.017) | (0.013) | (0.015) | (0.015) | (0.013) | (0.018) | (0.014) | (0.012) | (0.014) |
| Age squared | 0.003*** | -0.001*** | -0.001*** | -0.001*** | -0.001*** | 0.003*** | -0.001*** | -0.001*** | -0.001*** | -0.002*** |
|  | (0.000) | (0.000) | (0.000) | (0.000) | (0.000) | (0.000) | (0.000) | (0.000) | (0.000) | (0.000) |
| PC1 | -15.037*** | -2.777 | -4.848* | 5.284* | 18.622*** | -9.920*** | -4.869 | -0.463 | -0.219 | 13.590*** |
|  | (2.137) | (2.425) | (1.965) | (2.153) | (2.132) | (2.076) | (2.712) | (2.088) | (1.739) | (2.008) |
| PC2 | -2.153 | -1.713 | 1.273 | 1.451 | 0.237 | 3.564 | 0.984 | 2.510 | -2.229 | -3.750 |
|  | (2.395) | (2.701) | (1.951) | (2.039) | (2.040) | (2.332) | (2.589) | (2.208) | (1.760) | (1.966) |
| PC3 | 3.711 | -0.445 | -2.581 | -0.513 | -1.564 | 3.021 | -4.221 | 1.781 | 1.241 | -3.210 |
|  | (2.143) | (2.458) | (1.942) | (2.082) | (2.016) | (2.015) | (2.702) | (2.173) | (1.682) | (1.894) |
| Year dummies | Yes | Yes | Yes | Yes | Yes | Yes | Yes | Yes | Yes | Yes |
| Birth cohort dummies | Yes | Yes | Yes | Yes | Yes | Yes | Yes | Yes | Yes | Yes |
| Constant | 3.322*** | -4.574*** | -0.960** | -1.504*** | -3.620*** | 3.655*** | -3.597*** | -1.895*** | -1.381*** | -3.669*** |
|  | (0.351) | (0.422) | (0.329) | (0.363) | (0.362) | (0.326) | (0.456) | (0.345) | (0.284) | (0.341) |
|  |  |  |  |  |  |  |  |  |  |  |
| Pseudo-R^2^ | 0.131 | 0.021 | 0.024 | 0.020 | 0.031 | 0.130 | 0.015 | 0.011 | 0.024 | 0.038 |
| p(χ^2^) | *** | *** | *** | *** | *** | *** | *** | *** | *** | *** |

** P < 0.05, ** P < 0.005, *** P < 0.001; robust SE in parentheses; MDPGS denotes Mood Disorders Polygenic Score [22]*

**Supplementary Table S10** Differences in predicted probabilities of belonging to a specific labour market status in the DPGS, BDPGS and MDPGS top three deciles compared to the lowest decile in the pooled 1992-2017 sample

| **Sample** | **All** | **Males** | **Females** | **All** | **Males** | **Females** | **All** | **Males** | **Females** |
| --- | --- | --- | --- | --- | --- | --- | --- | --- | --- |
| **PGS** | **DPGS** | **DPGS** | **DPGS** | **BDPGS** | **BDPGS** | **BDPGS** | **MDPGS** | **MDPGS** | **MDPGS** |
| Total N in the model | 20,121 | 9,248 | 10,873 | 20,121 | 9,248 | 10,873 | 20,121 | 9,248 | 10,873 |
| **Reference: 1^st^ decile PGS** |  |  |  |  |  |  |  |  |  |
| Average marginal effects: |  |  |  |  |  |  |  |  |  |
| **8^th^ decile PGS** |  |  |  |  |  |  |  |  |  |
| Non-employed | 0.041*** | 0.025 | 0.054** | 0.029* | 0.031 | 0.026 | 0.042*** | 0.032 | 0.050** |
|  | (0.012) | (0.018) | (0.017) | (0.012) | (0.019) | (0.017) | (0.012) | (0.019) | (0.016) |
| Self-employed | -0.011 | -0.023 | -0.001 | 0.001 | 0.001 | 0.001 | -0.017 | -0.040* | 0.001 |
|  | (0.009) | (0.015) | (0.011) | (0.009) | (0.015) | (0.011) | (0.009) | (0.015) | (0.010) |
| Physical work | -0.012 | 0.003 | -0.025 | -0.052*** | -0.091*** | -0.021 | -0.022 | -0.004 | -0.036* |
|  | (0.013) | (0.020) | (0.016) | (0.013) | (0.020) | (0.016) | (0.012) | (0.021) | (0.015) |
| Office work | -0.007 | -0.004 | -0.010 | -0.016 | 0.008 | -0.039 | 0.001 | 0.016 | -0.013 |
|  | (0.014) | (0.017) | (0.021) | (0.014) | (0.017) | (0.021) | (0.014) | (0.017) | (0.021) |
| Knowledge work | -0.012 | -0.006 | -0.017 | 0.037** | 0.036* | 0.037* | 0.005 | -0.005 | 0.013 |
|  | (0.012) | (0.017) | (0.016) | (0.012) | (0.017) | (0.015) | (0.012) | (0.017) | (0.016) |
| **9^th^ decile PGS** |  |  |  |  |  |  |  |  |  |
| Non-employed | 0.049*** | 0.043* | 0.054** | 0.044*** | 0.035 | 0.052** | 0.047*** | 0.047* | 0.047** |
|  | (0.013) | (0.019) | (0.017) | (0.013) | (0.019) | (0.017) | (0.012) | (0.019) | (0.016) |
| Self-employed | -0.021* | -0.034* | -0.010 | -0.014 | -0.020 | -0.009 | -0.027** | -0.036* | -0.018 |
|  | (0.009) | (0.015) | (0.010) | (0.009) | (0.014) | (0.010) | (0.009) | (0.015) | (0.010) |
| Physical work | -0.006 | 0.009 | -0.021 | -0.054*** | -0.072*** | -0.039* | -0.003 | -0.023 | 0.011 |
|  | (0.013) | (0.020) | (0.016) | (0.013) | (0.020) | (0.015) | (0.013) | (0.020) | (0.016) |
| Office work | 0.001 | -0.008 | 0.011 | -0.009 | 0.005 | -0.021 | -0.003 | 0.003 | -0.009 |
|  | (0.014) | (0.017) | (0.021) | (0.014) | (0.017) | (0.020) | (0.014) | (0.017) | (0.021) |
| Knowledge work | -0.024* | -0.013 | -0.035* | 0.041*** | 0.058*** | 0.026 | -0.011 | -0.002 | -0.018 |
|  | (0.012) | (0.017) | (0.016) | (0.011) | (0.017) | (0.015) | (0.012) | (0.017) | (0.015) |
| **10^th^ decile PGS** |  |  |  |  |  |  |  |  |  |
| Non-employed | 0.069*** | 0.076*** | 0.064*** | 0.055*** | 0.064*** | 0.049** | 0.072*** | 0.067*** | 0.076*** |
|  | (0.013) | (0.019) | (0.017) | (0.013) | (0.019) | (0.017) | (0.013) | (0.019) | (0.017) |
| Self-employed | -0.020* | -0.042** | -0.003 | 0.001 | 0.001 | 0.000 | -0.013 | -0.039* | 0.009 |
|  | (0.009) | (0.015) | (0.011) | (0.009) | (0.015) | (0.011) | (0.009) | (0.015) | (0.011) |
| Physical work | -0.005 | 0.016 | -0.023 | -0.048*** | -0.075*** | -0.026 | -0.021 | -0.029 | -0.014 |
|  | (0.013) | (0.021) | (0.016) | (0.013) | (0.021) | (0.015) | (0.013) | (0.020) | (0.016) |
| Office work | 0.001 | -0.018 | 0.020 | -0.035* | -0.014 | -0.054* | -0.013 | 0.012 | -0.036 |
|  | (0.014) | (0.017) | (0.021) | (0.013) | (0.017) | (0.020) | (0.014) | (0.017) | (0.021) |
| Knowledge work | -0.047*** | -0.032 | -0.061*** | 0.033** | 0.022 | 0.042* | -0.016 | -0.005 | -0.027 |
|  | (0.011) | (0.017) | (0.015) | (0.011) | (0.017) | (0.016) | (0.012) | (0.017) | (0.015) |

** P < 0.05, ** P < 0.005, *** P < 0.001; robust SE in parentheses; polygenic scores (PGS) for depression (DPGS) [21], bipolar disorder (BDPGS) [18], and mood disorders (MDPGS) [22]; table presenting average marginal effects from probit models of labour market status with deciles of DPGS, BDPGS or MDPGS as main explanatory variable and controls for gender, age (linear and squared terms), birth cohort dummies, year dummies, and PC1-3.*

**Supplementary Table S11** Coefficient estimates from probit models of belonging to a specific labour market status within educational categories in the pooled 1992-2017 sample: Depression Polygenic Score (DPGS)

| **Sample** | **All with primary education** | **All with primary education** | **All with primary education** | **All with primary education** | **All with primary education** | **All with secondary education** | **All with secondary education** | **All with secondary education** | **All with secondary education** | **All with secondary education** | **All with higher education** | **All with higher education** | **All with higher education** | **All with higher education** | **All with higher education** |
| --- | --- | --- | --- | --- | --- | --- | --- | --- | --- | --- | --- | --- | --- | --- | --- |
| **PGS** | **DPGS** | **DPGS** | **DPGS** | **DPGS** | **DPGS** | **DPGS** | **DPGS** | **DPGS** | **DPGS** | **DPGS** | **DPGS** | **DPGS** | **DPGS** | **DPGS** | **DPGS** |
| **PGS specification** | **Linear** | **Linear** | **Linear** | **Linear** | **Linear** | **Linear** | **Linear** | **Linear** | **Linear** | **Linear** | **Linear** | **Linear** | **Linear** | **Linear** | **Linear** |
| **Labour market status** | **Non-employed** | **Self-employed** | **Physical**  **work** | **Office**  **work** | **Knowledge work** | **Non-employed** | **Self-employed** | **Physical**  **work** | **Office**  **work** | **Knowledge work** | **Non-employed** | **Self-employed** | **Physical**  **work** | **Office**  **work** | **Knowledge work** |
| N | 4,055 | 4,055 | 4,055 | 4,055 | 4,055 | 8,480 | 8,480 | 8,480 | 8,480 | 8,480 | 7,586 | 7,586 | 7,586 | 7,586 | 7,586 |
| Model | probit | probit | probit | probit | probit | probit | probit | probit | probit | probit | probit | probit | probit | probit | probit |
| **Dependent variable: belonging to the specific labour market status** | | | | | | | | | | |  |  |  |  |  |
| Explanatory variables: | | | | | | | | | | |  |  |  |  |  |
| PGS | 0.074* | -0.069 | -0.014 | -0.036 | 0.025 | 0.057* | -0.077** | -0.008 | 0.016 | -0.002 | 0.051 | -0.026 | 0.027 | -0.023 | -0.013 |
|  | (0.030) | (0.036) | (0.029) | (0.040) | (0.057) | (0.022) | (0.026) | (0.020) | (0.024) | (0.031) | (0.030) | (0.033) | (0.038) | (0.025) | (0.023) |
| Female | -0.068 | -0.347*** | -0.259*** | 0.713*** | -0.177* | -0.117*** | -0.253*** | -0.473*** | 0.803*** | -0.031 | -0.052 | -0.248*** | -0.166** | 0.425*** | -0.251*** |
|  | (0.043) | (0.056) | (0.043) | (0.052) | (0.090) | (0.031) | (0.038) | (0.029) | (0.032) | (0.048) | (0.038) | (0.046) | (0.053) | (0.031) | (0.030) |
| PGS # Female | -0.029 | 0.011 | 0.019 | 0.009 | -0.040 | 0.028 | 0.070 | -0.038 | -0.011 | -0.073 | 0.017 | 0.019 | -0.039 | 0.043 | -0.046 |
|  | (0.043) | (0.055) | (0.043) | (0.051) | (0.086) | (0.031) | (0.037) | (0.029) | (0.031) | (0.045) | (0.039) | (0.045) | (0.053) | (0.031) | (0.030) |
| Age | -0.205*** | 0.104** | 0.135*** | 0.070* | 0.142* | -0.238*** | 0.144*** | 0.069*** | 0.096*** | 0.077*** | -0.298*** | 0.124*** | -0.048 | 0.036* | 0.164*** |
|  | (0.026) | (0.035) | (0.026) | (0.031) | (0.068) | (0.014) | (0.017) | (0.013) | (0.014) | (0.021) | (0.017) | (0.024) | (0.025) | (0.014) | (0.014) |
| Age squared | 0.003*** | -0.001** | -0.002*** | -0.001** | -0.001* | 0.003*** | -0.002*** | -0.001*** | -0.001*** | -0.001*** | 0.004*** | -0.001*** | 0.001 | -0.001*** | -0.002*** |
|  | (0.000) | (0.000) | (0.000) | (0.000) | (0.001) | (0.000) | (0.000) | (0.000) | (0.000) | (0.000) | (0.000) | (0.000) | (0.000) | (0.000) | (0.000) |
| PC1 | -22.711*** | -5.521 | 9.498** | 13.258*** | 36.349*** | -5.639* | -4.484 | -1.136 | 5.769* | 12.712*** | -7.326* | 2.521 | -2.092 | -6.656** | 8.525*** |
|  | (3.115) | (3.910) | (3.064) | (3.534) | (6.974) | (2.250) | (2.732) | (2.050) | (2.151) | (3.430) | (2.663) | (3.073) | (3.637) | (2.115) | (2.042) |
| PC2 | 1.072 | 1.419 | -3.591 | 0.150 | -9.173 | 0.052 | -2.066 | 1.575 | 0.507 | 0.625 | 1.268 | -0.972 | 1.834 | -1.149 | 0.024 |
|  | (3.751) | (4.439) | (3.120) | (3.426) | (6.627) | (2.376) | (2.605) | (2.087) | (2.128) | (2.952) | (2.768) | (2.995) | (3.541) | (2.125) | (2.031) |
| PC3 | 3.427 | -4.465 | -0.725 | -0.302 | -1.140 | 5.298* | -1.504 | -0.718 | -3.287 | 0.186 | 0.095 | -1.183 | -0.547 | 4.084* | -3.205 |
|  | (3.140) | (4.233) | (3.013) | (3.339) | (5.434) | (2.124) | (2.593) | (2.003) | (2.019) | (2.978) | (2.600) | (3.001) | (3.936) | (2.060) | (2.015) |
| Year dummies | Yes | Yes | Yes | Yes | Yes | Yes | Yes | Yes | Yes | Yes | Yes | Yes | Yes | Yes | Yes |
| Birth cohort dummies | Yes | Yes | Yes | Yes | Yes | Yes | Yes | Yes | Yes | Yes | Yes | Yes | Yes | Yes | Yes |
| Constant | 2.704*** | -3.357*** | -2.492*** | -2.253** | -5.136** | 3.326*** | -4.294*** | -1.263*** | -2.535*** | -3.400*** | 4.320*** | -4.116*** | -0.737 | -0.973** | -3.552*** |
|  | (0.678) | (0.902) | (0.667) | (0.796) | (1.741) | (0.337) | (0.422) | (0.315) | (0.338) | (0.505) | (0.421) | (0.580) | (0.586) | (0.346) | (0.347) |
|  |  |  |  |  |  |  |  |  |  |  |  |  |  |  |  |
| Pseudo-R^2^ | 0.143 | 0.028 | 0.069 | 0.091 | 0.072 | 0.098 | 0.033 | 0.045 | 0.085 | 0.020 | 0.110 | 0.035 | 0.034 | 0.033 | 0.030 |
| p(χ^2^) | *** | *** | *** | *** | *** | *** | *** | *** | *** | *** | *** | *** | *** | *** | *** |

** P < 0.05, ** P < 0.005, *** P < 0.001; robust SE in parentheses; DPGS denotes Depression Polygenic Score [21]*

**Supplementary Table S12** Coefficient estimates from probit models of belonging to a specific labour market status within educational categories in the pooled 1992-2017 sample: Bipolar Disorder Polygenic Score (BDPGS)

| **Sample** | **All with primary education** | **All with primary education** | **All with primary education** | **All with primary education** | **All with primary education** | **All with secondary education** | **All with secondary education** | **All with secondary education** | **All with secondary education** | **All with secondary education** | **All with higher education** | **All with higher education** | **All with higher education** | **All with higher education** | **All with higher education** |
| --- | --- | --- | --- | --- | --- | --- | --- | --- | --- | --- | --- | --- | --- | --- | --- |
| **PGS** | **BDPGS** | **BDPGS** | **BDPGS** | **BDPGS** | **BDPGS** | **BDPGS** | **BDPGS** | **BDPGS** | **BDPGS** | **BDPGS** | **BDPGS** | **BDPGS** | **BDPGS** | **BDPGS** | **BDPGS** |
| **PGS specification** | **Linear** | **Linear** | **Linear** | **Linear** | **Linear** | **Linear** | **Linear** | **Linear** | **Linear** | **Linear** | **Linear** | **Linear** | **Linear** | **Linear** | **Linear** |
| **Labour market status** | **Non-employed** | **Self-employed** | **Physical**  **work** | **Office**  **work** | **Knowledge work** | **Non-employed** | **Self-employed** | **Physical**  **work** | **Office**  **work** | **Knowledge work** | **Non-employed** | **Self-employed** | **Physical**  **work** | **Office**  **work** | **Knowledge work** |
| N | 4,055 | 4,055 | 4,055 | 4,055 | 4,055 | 8,480 | 8,480 | 8,480 | 8,480 | 8,480 | 7,586 | 7,586 | 7,586 | 7,586 | 7,586 |
| Model | probit | probit | probit | probit | probit | probit | probit | probit | probit | probit | probit | probit | probit | probit | probit |
| **Dependent variable: belonging to the specific labour market status** | | | | | | | | | | |  |  |  |  |  |
| Explanatory variables: | | | | | | | | | | |  |  |  |  |  |
| PGS | 0.096** | -0.036 | -0.067* | -0.017 | 0.047 | 0.062* | -0.015 | -0.051* | 0.006 | 0.026 | 0.019 | 0.016 | -0.034 | -0.034 | 0.021 |
|  | (0.030) | (0.035) | (0.029) | (0.037) | (0.060) | (0.022) | (0.025) | (0.020) | (0.024) | (0.032) | (0.029) | (0.033) | (0.043) | (0.025) | (0.023) |
| Female | -0.072 | -0.345*** | -0.255*** | 0.713*** | -0.181* | -0.114*** | -0.256*** | -0.473*** | 0.803*** | -0.030 | -0.051 | -0.252*** | -0.164** | 0.423*** | -0.250*** |
|  | (0.043) | (0.056) | (0.043) | (0.052) | (0.090) | (0.031) | (0.038) | (0.029) | (0.032) | (0.048) | (0.038) | (0.046) | (0.053) | (0.031) | (0.030) |
| PGS # Female | -0.024 | 0.025 | 0.070 | -0.047 | 0.014 | -0.021 | 0.015 | 0.012 | -0.017 | 0.046 | 0.021 | 0.029 | -0.020 | -0.026 | 0.017 |
|  | (0.042) | (0.056) | (0.042) | (0.048) | (0.093) | (0.032) | (0.037) | (0.029) | (0.031) | (0.047) | (0.038) | (0.046) | (0.056) | (0.031) | (0.030) |
| Age | -0.202*** | 0.102** | 0.134*** | 0.070* | 0.147* | -0.238*** | 0.144*** | 0.069*** | 0.096*** | 0.078*** | -0.297*** | 0.125*** | -0.049 | 0.035* | 0.164*** |
|  | (0.026) | (0.035) | (0.026) | (0.031) | (0.067) | (0.014) | (0.017) | (0.013) | (0.014) | (0.021) | (0.017) | (0.024) | (0.025) | (0.014) | (0.014) |
| Age squared | 0.003*** | -0.001** | -0.002*** | -0.001** | -0.001* | 0.003*** | -0.002*** | -0.001*** | -0.001*** | -0.001*** | 0.004*** | -0.001*** | 0.001 | -0.001*** | -0.002*** |
|  | (0.000) | (0.000) | (0.000) | (0.000) | (0.001) | (0.000) | (0.000) | (0.000) | (0.000) | (0.000) | (0.000) | (0.000) | (0.000) | (0.000) | (0.000) |
| PC1 | -22.076*** | -5.179 | 8.902** | 12.741*** | 37.348*** | -5.918* | -3.896 | -1.380 | 5.574* | 14.078*** | -7.645** | 3.190 | -2.816 | -7.428*** | 9.538*** |
|  | (3.122) | (3.933) | (3.076) | (3.545) | (6.932) | (2.245) | (2.726) | (2.054) | (2.149) | (3.440) | (2.657) | (3.087) | (3.641) | (2.113) | (2.046) |
| PC2 | 0.374 | 1.638 | -3.335 | 0.640 | -10.002 | -0.056 | -2.027 | 1.718 | 0.508 | 0.563 | 1.214 | -1.209 | 2.024 | -0.871 | -0.264 |
|  | (3.697) | (4.397) | (3.148) | (3.432) | (6.531) | (2.357) | (2.595) | (2.093) | (2.128) | (2.927) | (2.810) | (2.958) | (3.602) | (2.130) | (2.044) |
| PC3 | 3.657 | -4.716 | -0.740 | -0.274 | -1.379 | 5.645* | -1.751 | -0.777 | -3.215 | -0.268 | 0.418 | -1.305 | -0.368 | 4.270* | -3.518 |
|  | (3.132) | (4.214) | (3.012) | (3.333) | (5.505) | (2.118) | (2.592) | (2.005) | (2.018) | (2.971) | (2.596) | (2.987) | (3.964) | (2.059) | (2.016) |
| Year dummies | Yes | Yes | Yes | Yes | Yes | Yes | Yes | Yes | Yes | Yes | Yes | Yes | Yes | Yes | Yes |
| Birth cohort dummies | Yes | Yes | Yes | Yes | Yes | Yes | Yes | Yes | Yes | Yes | Yes | Yes | Yes | Yes | Yes |
| Constant | 2.632*** | -3.314*** | -2.467*** | -2.244** | -5.271** | 3.313*** | -4.297*** | -1.262*** | -2.532*** | -3.414*** | 4.306*** | -4.132*** | -0.734 | -0.960* | -3.556*** |
|  | (0.680) | (0.900) | (0.667) | (0.796) | (1.724) | (0.337) | (0.422) | (0.315) | (0.338) | (0.506) | (0.421) | (0.580) | (0.588) | (0.346) | (0.347) |
|  |  |  |  |  |  |  |  |  |  |  |  |  |  |  |  |
| Pseudo-R^2^ | 0.145 | 0.027 | 0.070 | 0.092 | 0.073 | 0.097 | 0.031 | 0.046 | 0.085 | 0.020 | 0.109 | 0.035 | 0.035 | 0.034 | 0.029 |
| p(χ^2^) | *** | *** | *** | *** | *** | *** | *** | *** | *** | *** | *** | *** | *** | *** | *** |

** P < 0.05, ** P < 0.005, *** P < 0.001; robust SE in parentheses; DPGS denotes Bipolar Disorder Polygenic Score [18]*

**Supplementary Table S13** Coefficient estimates from probit models of belonging to a specific labour market status within educational categories in the pooled 1992-2017 sample: Mood Disorders Polygenic Score (MDPGS)

| **Sample** | **All with primary education** | **All with primary education** | **All with primary education** | **All with primary education** | **All with primary education** | **All with secondary education** | **All with secondary education** | **All with secondary education** | **All with secondary education** | **All with secondary education** | **All with higher education** | **All with higher education** | **All with higher education** | **All with higher education** | **All with higher education** |
| --- | --- | --- | --- | --- | --- | --- | --- | --- | --- | --- | --- | --- | --- | --- | --- |
| **PGS** | **MDPGS** | **MDPGS** | **MDPGS** | **MDPGS** | **MDPGS** | **MDPGS** | **MDPGS** | **MDPGS** | **MDPGS** | **MDPGS** | **MDPGS** | **MDPGS** | **MDPGS** | **MDPGS** | **MDPGS** |
| **PGS specification** | **Linear** | **Linear** | **Linear** | **Linear** | **Linear** | **Linear** | **Linear** | **Linear** | **Linear** | **Linear** | **Linear** | **Linear** | **Linear** | **Linear** | **Linear** |
| **Labour market status** | **Non-employed** | **Self-employed** | **Physical**  **work** | **Office**  **work** | **Knowledge work** | **Non-employed** | **Self-employed** | **Physical**  **work** | **Office**  **work** | **Knowledge work** | **Non-employed** | **Self-employed** | **Physical**  **work** | **Office**  **work** | **Knowledge work** |
| N | 4,055 | 4,055 | 4,055 | 4,055 | 4,055 | 8,480 | 8,480 | 8,480 | 8,480 | 8,480 | 7,586 | 7,586 | 7,586 | 7,586 | 7,586 |
| Model | probit | probit | probit | probit | probit | probit | probit | probit | probit | probit | probit | probit | probit | probit | probit |
| **Dependent variable: belonging to the specific labour market status** | | | | | | | | | | |  |  |  |  |  |
| Explanatory variables: | | | | | | | | | | |  |  |  |  |  |
| PGS | 0.053 | -0.053 | -0.017 | -0.046 | 0.106 | 0.099*** | -0.087*** | -0.037 | 0.023 | -0.010 | 0.038 | 0.003 | -0.001 | -0.010 | -0.016 |
|  | (0.030) | (0.036) | (0.029) | (0.038) | (0.061) | (0.022) | (0.025) | (0.020) | (0.024) | (0.031) | (0.030) | (0.032) | (0.043) | (0.025) | (0.023) |
| Female | -0.068 | -0.346*** | -0.263*** | 0.715*** | -0.180* | -0.114*** | -0.251*** | -0.473*** | 0.803*** | -0.027 | -0.053 | -0.250*** | -0.164** | 0.422*** | -0.249*** |
|  | (0.043) | (0.056) | (0.044) | (0.052) | (0.092) | (0.031) | (0.038) | (0.029) | (0.032) | (0.048) | (0.038) | (0.046) | (0.053) | (0.031) | (0.030) |
| PGS # Female | -0.034 | -0.004 | 0.083 | -0.004 | -0.014 | -0.009 | 0.105* | -0.015 | -0.045 | 0.011 | 0.047 | 0.005 | -0.025 | -0.005 | -0.014 |
|  | (0.042) | (0.055) | (0.043) | (0.049) | (0.089) | (0.032) | (0.038) | (0.029) | (0.031) | (0.045) | (0.039) | (0.045) | (0.056) | (0.032) | (0.030) |
| Age | -0.203*** | 0.103** | 0.135*** | 0.069* | 0.146* | -0.238*** | 0.143*** | 0.069*** | 0.096*** | 0.077*** | -0.298*** | 0.124*** | -0.048 | 0.036* | 0.164*** |
|  | (0.026) | (0.035) | (0.026) | (0.031) | (0.068) | (0.014) | (0.017) | (0.013) | (0.014) | (0.021) | (0.017) | (0.024) | (0.025) | (0.014) | (0.014) |
| Age squared | 0.003*** | -0.001** | -0.002*** | -0.001** | -0.001* | 0.003*** | -0.002*** | -0.001*** | -0.001*** | -0.001*** | 0.004*** | -0.001*** | 0.001 | -0.001*** | -0.002*** |
|  | (0.000) | (0.000) | (0.000) | (0.000) | (0.001) | (0.000) | (0.000) | (0.000) | (0.000) | (0.000) | (0.000) | (0.000) | (0.000) | (0.000) | (0.000) |
| PC1 | -23.213*** | -5.157 | 9.637** | 13.272*** | 37.197*** | -5.931* | -4.192 | -1.066 | 5.629* | 13.316*** | -7.745** | 2.841 | -2.289 | -6.759** | 8.911*** |
|  | (3.107) | (3.894) | (3.058) | (3.525) | (7.012) | (2.240) | (2.727) | (2.045) | (2.141) | (3.416) | (2.646) | (3.060) | (3.623) | (2.103) | (2.034) |
| PC2 | 0.962 | 1.583 | -3.638 | 0.292 | -10.102 | 0.031 | -2.010 | 1.581 | 0.485 | 0.626 | 1.266 | -1.044 | 1.825 | -1.096 | -0.028 |
|  | (3.752) | (4.425) | (3.134) | (3.431) | (6.470) | (2.361) | (2.606) | (2.089) | (2.127) | (2.942) | (2.778) | (2.966) | (3.577) | (2.121) | (2.033) |
| PC3 | 3.584 | -4.500 | -0.912 | -0.128 | -2.076 | 5.121* | -1.427 | -0.601 | -3.209 | -0.078 | 0.221 | -1.274 | -0.448 | 4.169* | -3.328 |
|  | (3.144) | (4.217) | (3.010) | (3.342) | (5.421) | (2.123) | (2.590) | (2.005) | (2.020) | (2.977) | (2.598) | (2.991) | (3.953) | (2.057) | (2.014) |
| Year dummies | Yes | Yes | Yes | Yes | Yes | Yes | Yes | Yes | Yes | Yes | Yes | Yes | Yes | Yes | Yes |
| Birth cohort dummies | Yes | Yes | Yes | Yes | Yes | Yes | Yes | Yes | Yes | Yes | Yes | Yes | Yes | Yes | Yes |
| Constant | 2.661*** | -3.347*** | -2.495*** | -2.227* | -5.251** | 3.328*** | -4.284*** | -1.261*** | -2.536*** | -3.401*** | 4.333*** | -4.119*** | -0.743 | -0.972** | -3.558*** |
|  | (0.678) | (0.901) | (0.668) | (0.797) | (1.744) | (0.337) | (0.423) | (0.315) | (0.338) | (0.504) | (0.421) | (0.580) | (0.587) | (0.346) | (0.347) |
|  |  |  |  |  |  |  |  |  |  |  |  |  |  |  |  |
| Pseudo-R^2^ | 0.142 | 0.028 | 0.069 | 0.092 | 0.077 | 0.099 | 0.034 | 0.046 | 0.085 | 0.019 | 0.110 | 0.035 | 0.034 | 0.033 | 0.029 |
| p(χ^2^) | *** | *** | *** | *** | *** | *** | *** | *** | *** | *** | *** | *** | *** | *** | *** |

** P < 0.05, ** P < 0.005, *** P < 0.001; robust standard errors (SE) in parentheses; MDPGS denotes Mood Disorders Polygenic Score [22]*

**Supplementary Table S14** Coefficient estimates from ordered probit regression models of equivalent income tertiles in the pooled 1992-2017 sample: Linear specification of polygenic scores

| **Sample** | **All** | **Males** | **Females** | **All** | **Males** | **Females** | **All** | **Males** | **Females** |
| --- | --- | --- | --- | --- | --- | --- | --- | --- | --- |
| **PGS** | **DPGS** | **DPGS** | **DPGS** | **BDPGS** | **BDPGS** | **BDPGS** | **MDPGS** | **MDPGS** | **MDPGS** |
| **PGS specification** | **Linear** | **Linear** | **Linear** | **Linear** | **Linear** | **Linear** | **Linear** | **Linear** | **Linear** |
| N | 19,685 | 9,098 | 10,587 | 19,685 | 9,098 | 10,587 | 19,685 | 9,098 | 10,587 |
| Estimation | oprobit | oprobit | oprobit | oprobit | oprobit | oprobit | oprobit | oprobit | oprobit |
| **Dependent variable: Belonging to a specific equivalent income tertile** | | | | | | | | | |
| Explanatory variables: |  |  |  |  |  |  |  |  |  |
| PGS | -0.038** | -0.035** | -0.039*** | -0.019 | -0.017 | 0.001 | -0.036** | -0.035** | -0.027* |
|  | (0.012) | (0.012) | (0.011) | (0.012) | (0.012) | (0.011) | (0.012) | (0.012) | (0.011) |
| Female | -0.087*** |  |  | -0.088*** |  |  | -0.087*** |  |  |
|  | (0.016) |  |  | (0.016) |  |  | (0.016) |  |  |
| PGS # Female | 0.001 |  |  | 0.022 |  |  | 0.010 |  |  |
|  | (0.016) |  |  | (0.016) |  |  | (0.016) |  |  |
| Age | 0.112*** | 0.121*** | 0.104*** | 0.111*** | 0.121*** | 0.104*** | 0.111*** | 0.121*** | 0.104*** |
|  | (0.007) | (0.011) | (0.010) | (0.007) | (0.011) | (0.010) | (0.007) | (0.011) | (0.010) |
| Age squared | -0.001*** | -0.001*** | -0.001*** | -0.001*** | -0.001*** | -0.001*** | -0.001*** | -0.001*** | -0.001*** |
|  | (0.000) | (0.000) | (0.000) | (0.000) | (0.000) | (0.000) | (0.000) | (0.000) | (0.000) |
| PC1 | 13.118*** | 15.268*** | 11.016*** | 13.558*** | 15.577*** | 11.563*** | 13.425*** | 15.505*** | 11.382*** |
|  | (1.157) | (1.696) | (1.587) | (1.157) | (1.693) | (1.591) | (1.151) | (1.686) | (1.581) |
| PC2 | -2.300 | -2.112 | -2.428 | -2.320 | -2.086 | -2.489 | -2.297 | -2.086 | -2.447 |
|  | (1.237) | (1.861) | (1.670) | (1.240) | (1.864) | (1.676) | (1.236) | (1.860) | (1.670) |
| PC3 | -3.786*** | -4.488* | -3.256* | -3.977*** | -4.638* | -3.478* | -3.824*** | -4.486* | -3.330* |
|  | (1.125) | (1.675) | (1.522) | (1.125) | (1.674) | (1.523) | (1.125) | (1.675) | (1.523) |
| Year dummies | Yes | Yes | Yes | Yes | Yes | Yes | Yes | Yes | Yes |
| Birth cohort dummies | Yes | Yes | Yes | Yes | Yes | Yes | Yes | Yes | Yes |
|  |  |  |  |  |  |  |  |  |  |
| Cut: Lowest/Medium | 1.718*** | 2.034*** | 1.557*** | 1.714*** | 2.027*** | 1.556*** | 1.717*** | 2.030*** | 1.558*** |
|  | (0.184) | (0.272) | (0.250) | (0.184) | (0.272) | (0.250) | (0.184) | (0.272) | (0.250) |
| Cut: Medium/Highest | 2.652*** | 2.931*** | 2.523*** | 2.647*** | 2.924*** | 2.521*** | 2.650*** | 2.927*** | 2.523*** |
|  | (0.184) | (0.273) | (0.250) | (0.184) | (0.272) | (0.250) | (0.184) | (0.272) | (0.250) |
|  |  |  |  |  |  |  |  |  |  |
| Pseudo-R^2^ | 0.022 | 0.022 | 0.021 | 0.021 | 0.021 | 0.021 | 0.022 | 0.022 | 0.021 |
| p(χ^2^) | *** | *** | *** | *** | *** | *** | *** | *** | *** |

** P < 0.05, ** P < 0.005, *** P < 0.001; robust SE in parentheses; polygenic scores (PGS) for depression (DPGS) [21], bipolar disorder (BDPGS) [18], and mood disorders (MDPGS) [22].*

**Supplementary Table S15** Differences in predicted probabilities of belonging to a specific equivalent income tertile in the DPGS and MDPGS top three deciles compared to the lowest decile in the pooled 1992-2017 sample

| **Sample** | **All** | **Males** | **Females** | **All** | **Males** | **Females** |
| --- | --- | --- | --- | --- | --- | --- |
| **PGS** | **DPGS** | **DPGS** | **DPGS** | **MDPGS** | **MDPGS** | **MDPGS** |
| Total N in the model | 19,685 | 9,098 | 10,587 | 19,685 | 9,098 | 10,587 |
| **Reference: 1^st^ decile PGS** |  |  |  |  |  |  |
| Average marginal effects: |  |  |  |  |  |  |
| **8^th^ decile PGS** |  |  |  |  |  |  |
| Lowest equivalent income tertile | 0.020 | 0.031 | 0.009 | 0.022 | 0.025 | 0.017 |
|  | (0.013) | (0.018) | (0.018) | (0.013) | (0.018) | (0.017) |
| Medium equivalent income tertile | -0.000 | 0.001 | -0.000 | 0.001 | 0.003 | -0.001 |
|  | (0.000) | (0.001) | (0.001) | (0.000) | (0.002) | (0.001) |
| Highest equivalent income tertile | -0.020 | -0.032 | -0.009 | -0.023 | -0.028 | -0.016 |
|  | (0.013) | (0.019) | (0.017) | (0.013) | (0.020) | (0.017) |
| **9^th^ decile PGS** |  |  |  |  |  |  |
| Lowest equivalent income tertile | 0.021 | 0.010 | 0.031 | 0.039** | 0.057** | 0.021 |
|  | (0.013) | (0.018) | (0.018) | (0.013) | (0.018) | (0.017) |
| Medium equivalent income tertile | -0.000 | 0.001 | -0.002 | 0.000 | 0.004* | -0.001 |
|  | (0.000) | (0.001) | (0.001) | (0.001) | (0.002) | (0.001) |
| Highest equivalent income tertile | -0.021 | -0.011 | -0.029 | -0.039** | -0.061** | -0.020 |
|  | (0.013) | (0.019) | (0.017) | (0.013) | (0.019) | (0.016) |
| **10^th^ decile PGS** |  |  |  |  |  |  |
| Lowest equivalent income tertile | 0.046*** | 0.069*** | 0.026 | 0.049*** | 0.069*** | 0.029 |
|  | (0.013) | (0.019) | (0.018) | (0.013) | (0.018) | (0.018) |
| Medium equivalent income tertile | -0.001 | -0.000 | -0.002 | -0.000 | 0.003 | -0.002 |
|  | (0.001) | (0.002) | (0.001) | (0.001) | (0.002) | (0.001) |
| Highest equivalent income tertile | -0.044*** | -0.069*** | -0.024 | -0.049*** | -0.073*** | -0.027 |
|  | (0.012) | (0.019) | (0.017) | (0.012) | (0.019) | (0.017) |

** P < 0.05, ** P < 0.005, *** P < 0.001; robust SE in parentheses; polygenic scores (PGS) for depression (DPGS) [21] and mood disorders (MDPGS) [22]; table presenting average marginal effects from ordered probit models of equivalent income tertile with deciles of DPGS or MDPGS as main explanatory variable and controls for gender, birth cohort dummies and PC1-3.*

**Supplementary Table S16** Coefficient estimates from ordered probit regression models of equivalent income tertiles in the pooled 1992-2017 sample: Non-linear specification of polygenic scores

| **Sample** | **All** | **Males** | **Females** | **All** | **Males** | **Females** | **All** | **Males** | **Females** |
| --- | --- | --- | --- | --- | --- | --- | --- | --- | --- |
| **PGS** | **DPGS** | **DPGS** | **DPGS** | **BDPGS** | **BDPGS** | **BDPGS** | **MDPGS** | **MDPGS** | **MDPGS** |
| **PGS specification** | **Linear + squared** | **Linear + squared** | **Linear + squared** | **Linear + squared** | **Linear + squared** | **Linear + squared** | **Linear + squared** | **Linear + squared** | **Linear + squared** |
| N | 19,685 | 9,098 | 10,587 | 19,685 | 9,098 | 10,587 | 19,685 | 9,098 | 10,587 |
| Estimation | oprobit | oprobit | oprobit | oprobit | oprobit | oprobit | oprobit | oprobit | oprobit |
| **Dependent variable: Belonging to a specific equivalent income tertile** | | | | | | | | | |
| Explanatory variables: |  |  |  |  |  |  |  |  |  |
| PGS | -0.037*** | -0.036** | -0.039*** | -0.008 | -0.018 | -0.000 | -0.031*** | -0.035** | -0.027* |
|  | (0.008) | (0.012) | (0.011) | (0.008) | (0.012) | (0.011) | (0.008) | (0.012) | (0.011) |
| PGS squared | -0.005 | -0.014 | 0.002 | -0.017** | -0.010 | -0.022** | -0.005 | -0.009 | -0.002 |
|  | (0.006) | (0.008) | (0.008) | (0.006) | (0.009) | (0.008) | (0.006) | (0.008) | (0.008) |
| Female | -0.087*** |  |  | -0.088*** |  |  | -0.087*** |  |  |
|  | (0.016) |  |  | (0.016) |  |  | (0.016) |  |  |
| Age | 0.112*** | 0.121*** | 0.104*** | 0.111*** | 0.121*** | 0.104*** | 0.111*** | 0.121*** | 0.104*** |
|  | (0.007) | (0.011) | (0.010) | (0.007) | (0.011) | (0.010) | (0.007) | (0.011) | (0.010) |
| Age squared | -0.001*** | -0.001*** | -0.001*** | -0.001*** | -0.001*** | -0.001*** | -0.001*** | -0.001*** | -0.001*** |
|  | (0.000) | (0.000) | (0.000) | (0.000) | (0.000) | (0.000) | (0.000) | (0.000) | (0.000) |
| PC1 | 13.148*** | 15.330*** | 11.006*** | 13.690*** | 15.646*** | 11.751*** | 13.455*** | 15.522*** | 11.391*** |
|  | (1.157) | (1.696) | (1.588) | (1.158) | (1.692) | (1.594) | (1.151) | (1.685) | (1.582) |
| PC2 | -2.300 | -2.087 | -2.426 | -2.244 | -2.008 | -2.435 | -2.303 | -2.067 | -2.452 |
|  | (1.237) | (1.862) | (1.670) | (1.234) | (1.853) | (1.673) | (1.236) | (1.857) | (1.671) |
| PC3 | -3.768*** | -4.431* | -3.260* | -3.964*** | -4.647* | -3.460* | -3.815*** | -4.448* | -3.331* |
|  | (1.125) | (1.676) | (1.523) | (1.124) | (1.672) | (1.523) | (1.125) | (1.675) | (1.523) |
| Year dummies | Yes | Yes | Yes | Yes | Yes | Yes | Yes | Yes | Yes |
| Birth cohort dummies | Yes | Yes | Yes | Yes | Yes | Yes | Yes | Yes | Yes |
|  |  |  |  |  |  |  |  |  |  |
| Cut: Lowest/Medium | 1.713*** | 2.020*** | 1.558*** | 1.697*** | 2.017*** | 1.532*** | 1.713*** | 2.025*** | 1.556*** |
|  | (0.184) | (0.272) | (0.250) | (0.184) | (0.272) | (0.250) | (0.184) | (0.272) | (0.250) |
| Cut: Medium/Highest | 2.647*** | 2.918*** | 2.524*** | 2.630*** | 2.915*** | 2.497*** | 2.646*** | 2.922*** | 2.522*** |
|  | (0.184) | (0.273) | (0.250) | (0.184) | (0.273) | (0.250) | (0.184) | (0.272) | (0.250) |
|  |  |  |  |  |  |  |  |  |  |
| Pseudo-R^2^ | 0.022 | 0.022 | 0.021 | 0.021 | 0.021 | 0.021 | 0.022 | 0.022 | 0.021 |
| p(χ^2^) | *** | *** | *** | *** | *** | *** | *** | *** | *** |

** P < 0.05, ** P < 0.005, *** P < 0.001; robust SE in parentheses; polygenic scores (PGS) for depression (DPGS) [21], bipolar disorder (BDPGS) [18], and mood disorders (MDPGS) [22].*

**Supplementary Table S17** Coefficient estimates from ordered probit regression models of equivalent income tertiles within educational categories in the pooled 1992-2017 sample: Depression Polygenic Score (DPGS)

| **Sample** | **All with primary education** | **Males with primary education** | **Females with primary education** | **All with secondary education** | **Males with secondary education** | **Females with secondary education** | **All with higher education** | **Males with higher education** | **Females with higher education** |
| --- | --- | --- | --- | --- | --- | --- | --- | --- | --- |
| **PGS** | **DPGS** | **DPGS** | **DPGS** | **DPGS** | **DPGS** | **DPGS** | **DPGS** | **DPGS** | **DPGS** |
| **PGS specification** | **Linear** | **Linear** | **Linear** | **Linear** | **Linear** | **Linear** | **Linear** | **Linear** | **Linear** |
| N | 3,911 | 1,995 | 1,916 | 8,276 | 4,075 | 4,201 | 7,498 | 3,028 | 4,470 |
| Estimation | oprobit | oprobit | oprobit | oprobit | oprobit | oprobit | oprobit | oprobit | oprobit |
| **Dependent variable: Belonging to a specific equivalent income tertile** | | | | | | | | | |
| Explanatory variables: |  |  |  |  |  |  |  |  |  |
| PGS | -0.032 | -0.034 | -0.007 | -0.013 | -0.008 | -0.027 | -0.050* | -0.051* | -0.042* |
|  | (0.026) | (0.026) | (0.027) | (0.018) | (0.018) | (0.018) | (0.021) | (0.021) | (0.017) |
| Female | -0.143*** |  |  | -0.152*** |  |  | -0.171*** |  |  |
|  | (0.037) |  |  | (0.025) |  |  | (0.028) |  |  |
| PGS # Female | 0.021 |  |  | -0.010 |  |  | 0.008 |  |  |
|  | (0.037) |  |  | (0.025) |  |  | (0.027) |  |  |
| Age | 0.135*** | 0.138*** | 0.122*** | 0.101*** | 0.108*** | 0.094*** | 0.105*** | 0.130*** | 0.097*** |
|  | (0.024) | (0.031) | (0.036) | (0.011) | (0.016) | (0.016) | (0.013) | (0.021) | (0.016) |
| Age squared | -0.002*** | -0.002*** | -0.001*** | -0.001*** | -0.001*** | -0.001*** | -0.001*** | -0.001*** | -0.001*** |
|  | (0.000) | (0.000) | (0.000) | (0.000) | (0.000) | (0.000) | (0.000) | (0.000) | (0.000) |
| PC1 | 14.324*** | 11.444** | 17.474*** | 10.230*** | 13.919*** | 5.959* | 7.706*** | 7.035* | 8.111*** |
|  | (2.659) | (3.559) | (4.028) | (1.828) | (2.591) | (2.615) | (1.871) | (3.039) | (2.385) |
| PC2 | -1.547 | -1.780 | -0.126 | -0.632 | -2.313 | 1.425 | -2.232 | -1.011 | -2.925 |
|  | (2.849) | (4.124) | (3.913) | (1.928) | (2.857) | (2.609) | (1.944) | (3.011) | (2.603) |
| PC3 | -5.249* | -5.405 | -5.817 | -4.041* | -4.105 | -4.446 | -2.766 | -3.748 | -2.314 |
|  | (2.650) | (3.824) | (3.721) | (1.690) | (2.391) | (2.425) | (1.853) | (3.041) | (2.349) |
| Year dummies | Yes | Yes | Yes | Yes | Yes | Yes | Yes | Yes | Yes |
| Birth cohort dummies | Yes | Yes | Yes | Yes | Yes | Yes | Yes | Yes | Yes |
|  |  |  |  |  |  |  |  |  |  |
| Cut: Lowest/Medium | 2.417*** | 2.499** | 2.230* | 1.646*** | 1.785*** | 1.617*** | 1.320*** | 2.055*** | 1.184** |
|  | (0.609) | (0.794) | (0.945) | (0.269) | (0.379) | (0.383) | (0.304) | (0.509) | (0.379) |
| Cut: Medium/Highest | 3.408*** | 3.461*** | 3.263*** | 2.685*** | 2.775*** | 2.709*** | 2.293*** | 2.989*** | 2.183*** |
|  | (0.610) | (0.795) | (0.946) | (0.270) | (0.380) | (0.384) | (0.305) | (0.510) | (0.380) |
|  |  |  |  |  |  |  |  |  |  |
| Pseudo-R^2^ | 0.029 | 0.024 | 0.037 | 0.024 | 0.022 | 0.026 | 0.033 | 0.031 | 0.029 |
| p(χ^2^) | *** | *** | *** | *** | *** | *** | *** | *** | *** |

** P < 0.05, ** P < 0.005, *** P < 0.001; robust SE in parentheses; polygenic score (PGS) for depression (DPGS) [21].*

**Supplementary Table S18** Coefficient estimates from ordered probit regression models of equivalent income tertiles within educational categories in the pooled 1992-2017 sample: Bipolar Disorder Polygenic Score (BDPGS)

| **Sample** | **All with primary education** | **Males with primary education** | **Females with primary education** | **All with secondary education** | **Males with secondary education** | **Females with secondary education** | **All with higher education** | **Males with higher education** | **Females with higher education** |
| --- | --- | --- | --- | --- | --- | --- | --- | --- | --- |
| **PGS** | **BDPGS** | **BDPGS** | **BDPGS** | **BDPGS** | **BDPGS** | **BDPGS** | **BDPGS** | **BDPGS** | **BDPGS** |
| **PGS specification** | **Linear** | **Linear** | **Linear** | **Linear** | **Linear** | **Linear** | **Linear** | **Linear** | **Linear** |
| N | 3,911 | 1,995 | 1,916 | 8,276 | 4,075 | 4,201 | 7,498 | 3,028 | 4,470 |
| Estimation | oprobit | oprobit | oprobit | oprobit | oprobit | oprobit | oprobit | oprobit | oprobit |
| **Dependent variable: Belonging to a specific equivalent income tertile** | | | | | | | | | |
| Explanatory variables: |  |  |  |  |  |  |  |  |  |
| PGS | -0.001 | -0.002 | -0.022 | -0.070*** | -0.066*** | 0.005 | 0.010 | 0.008 | -0.012 |
|  | (0.026) | (0.026) | (0.027) | (0.018) | (0.018) | (0.018) | (0.022) | (0.022) | (0.017) |
| Female | -0.142*** |  |  | -0.149*** |  |  | -0.172*** |  |  |
|  | (0.037) |  |  | (0.025) |  |  | (0.028) |  |  |
| PGS # Female | -0.024 |  |  | 0.078** |  |  | -0.022 |  |  |
|  | (0.036) |  |  | (0.025) |  |  | (0.028) |  |  |
| Age | 0.134*** | 0.137*** | 0.122*** | 0.102*** | 0.108*** | 0.094*** | 0.105*** | 0.129*** | 0.097*** |
|  | (0.024) | (0.031) | (0.036) | (0.011) | (0.016) | (0.016) | (0.013) | (0.021) | (0.016) |
| Age squared | -0.002*** | -0.002*** | -0.001*** | -0.001*** | -0.001*** | -0.001*** | -0.001*** | -0.001*** | -0.001*** |
|  | (0.000) | (0.000) | (0.000) | (0.000) | (0.000) | (0.000) | (0.000) | (0.000) | (0.000) |
| PC1 | 14.386*** | 11.900*** | 17.116*** | 10.044*** | 13.118*** | 6.474* | 8.325*** | 8.120* | 8.439*** |
|  | (2.672) | (3.577) | (4.052) | (1.822) | (2.574) | (2.614) | (1.869) | (3.018) | (2.391) |
| PC2 | -1.456 | -1.882 | 0.118 | -0.496 | -2.103 | 1.432 | -2.297 | -1.136 | -2.954 |
|  | (2.860) | (4.149) | (3.923) | (1.910) | (2.802) | (2.614) | (1.965) | (3.081) | (2.610) |
| PC3 | -5.277* | -5.562 | -5.730 | -4.099* | -4.014 | -4.630 | -2.991 | -4.001 | -2.511 |
|  | (2.653) | (3.825) | (3.725) | (1.688) | (2.383) | (2.424) | (1.853) | (3.038) | (2.350) |
| Year dummies | Yes | Yes | Yes | Yes | Yes | Yes | Yes | Yes | Yes |
| Birth cohort dummies | Yes | Yes | Yes | Yes | Yes | Yes | Yes | Yes | Yes |
|  |  |  |  |  |  |  |  |  |  |
| Cut: Lowest/Medium | 2.396*** | 2.456** | 2.235* | 1.652*** | 1.790*** | 1.618*** | 1.315*** | 2.043*** | 1.184** |
|  | (0.608) | (0.793) | (0.944) | (0.269) | (0.378) | (0.383) | (0.304) | (0.508) | (0.379) |
| Cut: Medium/Highest | 3.388*** | 3.418*** | 3.269*** | 2.692*** | 2.783*** | 2.709*** | 2.287*** | 2.976*** | 2.182*** |
|  | (0.609) | (0.794) | (0.946) | (0.270) | (0.380) | (0.384) | (0.305) | (0.509) | (0.380) |
|  |  |  |  |  |  |  |  |  |  |
| Pseudo-R^2^ | 0.029 | 0.024 | 0.038 | 0.025 | 0.023 | 0.025 | 0.032 | 0.030 | 0.028 |
| p(χ^2^) | *** | *** | *** | *** | *** | *** | *** | *** | *** |

** P < 0.05, ** P < 0.005, *** P < 0.001; robust SE in parentheses; polygenic score (PGS) for bipolar disorder (BDPGS) [18].*

**Supplementary Table S19** Coefficient estimates from ordered probit regression models of equivalent income tertiles within educational categories in the pooled 1992-2017 sample: Bipolar Disorder Polygenic Score (BDPGS)

| **Sample** | **All with primary education** | **Males with primary education** | **Females with primary education** | **All with secondary education** | **Males with secondary education** | **Females with secondary education** | **All with higher education** | **Males with higher education** | **Females with higher education** |
| --- | --- | --- | --- | --- | --- | --- | --- | --- | --- |
| **PGS** | **BDPGS** | **BDPGS** | **BDPGS** | **BDPGS** | **BDPGS** | **BDPGS** | **BDPGS** | **BDPGS** | **BDPGS** |
| **PGS specification** | **Linear + squared** | **Linear + squared** | **Linear + squared** | **Linear + squared** | **Linear + squared** | **Linear + squared** | **Linear + squared** | **Linear + squared** | **Linear + squared** |
| N | 3,911 | 1,995 | 1,916 | 8,276 | 4,075 | 4,201 | 7,498 | 3,028 | 4,470 |
| Estimation | oprobit | oprobit | oprobit | oprobit | oprobit | oprobit | oprobit | oprobit | oprobit |
| **Dependent variable: Belonging to a specific equivalent income tertile** | | | | | | | | | |
| Explanatory variables: |  |  |  |  |  |  |  |  |  |
| PGS | -0.015 | -0.004 | -0.026 | -0.031* | -0.065*** | 0.003 | -0.004 | 0.006 | -0.013 |
|  | (0.019) | (0.026) | (0.027) | (0.013) | (0.018) | (0.018) | (0.013) | (0.022) | (0.017) |
| PGS squared | -0.019 | -0.014 | -0.029 | -0.007 | 0.005 | -0.018 | -0.022* | -0.027 | -0.018 |
|  | (0.013) | (0.018) | (0.019) | (0.009) | (0.012) | (0.012) | (0.010) | (0.016) | (0.012) |
| Female | -0.143*** |  |  | -0.152*** |  |  | -0.173*** |  |  |
|  | (0.037) |  |  | (0.025) |  |  | (0.028) |  |  |
| Age | 0.133*** | 0.136*** | 0.121*** | 0.102*** | 0.108*** | 0.094*** | 0.105*** | 0.130*** | 0.097*** |
|  | (0.024) | (0.031) | (0.037) | (0.011) | (0.016) | (0.016) | (0.013) | (0.021) | (0.016) |
| Age squared | -0.002*** | -0.002*** | -0.001*** | -0.001*** | -0.001*** | -0.001*** | -0.001*** | -0.001*** | -0.001*** |
|  | (0.000) | (0.000) | (0.000) | (0.000) | (0.000) | (0.000) | (0.000) | (0.000) | (0.000) |
| PC1 | 14.566*** | 12.002*** | 17.494*** | 10.132*** | 13.085*** | 6.668* | 8.443*** | 8.267* | 8.542*** |
|  | (2.674) | (3.574) | (4.069) | (1.824) | (2.575) | (2.620) | (1.869) | (3.011) | (2.394) |
| PC2 | -1.264 | -1.558 | 0.070 | -0.572 | -2.117 | 1.411 | -2.163 | -0.967 | -2.841 |
|  | (2.816) | (4.082) | (3.910) | (1.916) | (2.806) | (2.615) | (1.954) | (3.045) | (2.608) |
| PC3 | -5.216* | -5.501 | -5.557 | -4.082* | -4.008 | -4.652 | -3.019 | -4.072 | -2.498 |
|  | (2.648) | (3.816) | (3.720) | (1.688) | (2.384) | (2.423) | (1.852) | (3.031) | (2.351) |
| Year dummies | Yes | Yes | Yes | Yes | Yes | Yes | Yes | Yes | Yes |
| Birth cohort dummies | Yes | Yes | Yes | Yes | Yes | Yes | Yes | Yes | Yes |
|  |  |  |  |  |  |  |  |  |  |
| Cut: Lowest/Medium | 2.348*** | 2.428** | 2.170* | 1.641*** | 1.794*** | 1.607*** | 1.289*** | 2.024*** | 1.156** |
|  | (0.610) | (0.795) | (0.947) | (0.269) | (0.379) | (0.383) | (0.305) | (0.509) | (0.380) |
| Cut: Medium/Highest | 3.340*** | 3.390*** | 3.205*** | 2.680*** | 2.786*** | 2.699*** | 2.261*** | 2.957*** | 2.154*** |
|  | (0.611) | (0.796) | (0.948) | (0.270) | (0.380) | (0.384) | (0.305) | (0.510) | (0.381) |
|  |  |  |  |  |  |  |  |  |  |
| Pseudo-R^2^ | 0.029 | 0.024 | 0.038 | 0.025 | 0.023 | 0.026 | 0.032 | 0.031 | 0.028 |
| p(χ^2^) | *** | *** | *** | *** | *** | *** | *** | *** | *** |

** P < 0.05, ** P < 0.005, *** P < 0.001; robust SE in parentheses; polygenic score (PGS) for bipolar disorder (BDPGS) [18].*

**Supplementary Table S20** Coefficient estimates from ordered probit regression models of equivalent income tertiles within educational categories in the pooled 1992-2017 sample: Mood Disorders Polygenic Score (MDPGS)

| **Sample** | **All with primary education** | **Males with primary education** | **Females with primary education** | **All with secondary education** | **Males with secondary education** | **Females with secondary education** | **All with higher education** | **Males with higher education** | **Females with higher education** |
| --- | --- | --- | --- | --- | --- | --- | --- | --- | --- |
| **PGS** | **MDPGS** | **MDPGS** | **MDPGS** | **MDPGS** | **MDPGS** | **MDPGS** | **MDPGS** | **MDPGS** | **MDPGS** |
| **PGS specification** | **Linear** | **Linear** | **Linear** | **Linear** | **Linear** | **Linear** | **Linear** | **Linear** | **Linear** |
| N | 3,911 | 1,995 | 1,916 | 8,276 | 4,075 | 4,201 | 7,498 | 3,028 | 4,470 |
| Estimation | oprobit | oprobit | oprobit | oprobit | oprobit | oprobit | oprobit | oprobit | oprobit |
| **Dependent variable: Belonging to a specific equivalent income tertile** | | | | | | | | | |
| Explanatory variables: |  |  |  |  |  |  |  |  |  |
| PGS | -0.011 | -0.014 | -0.000 | -0.062*** | -0.058*** | -0.023 | -0.030 | -0.032 | -0.033 |
|  | (0.026) | (0.026) | (0.028) | (0.018) | (0.018) | (0.017) | (0.022) | (0.022) | (0.017) |
| Female | -0.142*** |  |  | -0.151*** |  |  | -0.172*** |  |  |
|  | (0.037) |  |  | (0.025) |  |  | (0.028) |  |  |
| PGS # Female | 0.007 |  |  | 0.041 |  |  | -0.002 |  |  |
|  | (0.037) |  |  | (0.025) |  |  | (0.028) |  |  |
| Age | 0.134*** | 0.137*** | 0.122*** | 0.101*** | 0.108*** | 0.094*** | 0.105*** | 0.130*** | 0.097*** |
|  | (0.024) | (0.031) | (0.036) | (0.011) | (0.016) | (0.016) | (0.013) | (0.021) | (0.016) |
| Age squared | -0.002*** | -0.002*** | -0.001*** | -0.001*** | -0.001*** | -0.001*** | -0.001*** | -0.001*** | -0.001*** |
|  | (0.000) | (0.000) | (0.000) | (0.000) | (0.000) | (0.000) | (0.000) | (0.000) | (0.000) |
| PC1 | 14.548*** | 11.790*** | 17.554*** | 10.137*** | 13.494*** | 6.250* | 8.151*** | 7.676* | 8.441*** |
|  | (2.652) | (3.550) | (4.016) | (1.816) | (2.572) | (2.600) | (1.860) | (3.008) | (2.376) |
| PC2 | -1.560 | -1.795 | -0.138 | -0.621 | -2.286 | 1.405 | -2.243 | -1.014 | -2.951 |
|  | (2.856) | (4.143) | (3.914) | (1.921) | (2.830) | (2.611) | (1.950) | (3.038) | (2.602) |
| PC3 | -5.305* | -5.507 | -5.846 | -3.895* | -3.783 | -4.487 | -2.874 | -3.863 | -2.415 |
|  | (2.652) | (3.826) | (3.725) | (1.690) | (2.390) | (2.425) | (1.851) | (3.035) | (2.348) |
| Year dummies | Yes | Yes | Yes | Yes | Yes | Yes | Yes | Yes | Yes |
| Birth cohort dummies | Yes | Yes | Yes | Yes | Yes | Yes | Yes | Yes | Yes |
|  |  |  |  |  |  |  |  |  |  |
| Cut: Lowest/Medium | 2.395*** | 2.463** | 2.226* | 1.643*** | 1.776*** | 1.617*** | 1.325*** | 2.058*** | 1.192** |
|  | (0.609) | (0.793) | (0.945) | (0.269) | (0.379) | (0.383) | (0.304) | (0.508) | (0.379) |
| Cut: Medium/Highest | 3.387*** | 3.425*** | 3.260*** | 2.682*** | 2.768*** | 2.709*** | 2.297*** | 2.991*** | 2.190*** |
|  | (0.610) | (0.794) | (0.946) | (0.270) | (0.380) | (0.384) | (0.305) | (0.510) | (0.380) |
|  |  |  |  |  |  |  |  |  |  |
| Pseudo-R^2^ | 0.029 | 0.024 | 0.037 | 0.025 | 0.023 | 0.026 | 0.032 | 0.031 | 0.028 |
| p(χ^2^) | *** | *** | *** | *** | *** | *** | *** | *** | *** |

** P < 0.05, ** P < 0.005, *** P < 0.001; robust SE in parentheses; polygenic score (PGS) for mood disorders (MDPGS) [22].*

**Supplementary Table S21** Coefficient estimates from probit regression models of economic satisfaction in the pooled 1992-2017 sample: Linear specification of polygenic scores

| **Sample** | **All** | **Males** | **Females** | **All** | **Males** | **Females** | **All** | **Males** | **Females** |
| --- | --- | --- | --- | --- | --- | --- | --- | --- | --- |
| **PGS** | **DPGS** | **DPGS** | **DPGS** | **BDPGS** | **BDPGS** | **BDPGS** | **MDPGS** | **MDPGS** | **MDPGS** |
| **PGS specification** | **Linear** | **Linear** | **Linear** | **Linear** | **Linear** | **Linear** | **Linear** | **Linear** | **Linear** |
| N | 20,029 | 9,201 | 10,828 | 20,029 | 9,201 | 10,828 | 20,029 | 9,201 | 10,828 |
| Estimation | probit | probit | probit | probit | probit | probit | probit | probit | probit |
| **Dependent variable: Economic satisfaction** | | | | | | | | | |
| Explanatory variables: |  |  |  |  |  |  |  |  |  |
| PGS | -0.059*** | -0.057*** | -0.056*** | -0.004 | -0.003 | -0.023 | -0.047** | -0.046** | -0.057*** |
|  | (0.016) | (0.016) | (0.015) | (0.015) | (0.016) | (0.015) | (0.015) | (0.015) | (0.015) |
| Female | 0.074*** |  |  | 0.073*** |  |  | 0.075*** |  |  |
|  | (0.021) |  |  | (0.021) |  |  | (0.021) |  |  |
| PGS # Female | 0.003 |  |  | -0.019 |  |  | -0.010 |  |  |
|  | (0.021) |  |  | (0.021) |  |  | (0.021) |  |  |
| Age | 0.022* | 0.025 | 0.019 | 0.021* | 0.024 | 0.018 | 0.022* | 0.024 | 0.019 |
|  | (0.010) | (0.014) | (0.014) | (0.010) | (0.014) | (0.014) | (0.010) | (0.014) | (0.014) |
| Age squared | -0.000 | -0.000 | -0.000 | -0.000 | -0.000 | -0.000 | -0.000 | -0.000 | -0.000 |
|  | (0.000) | (0.000) | (0.000) | (0.000) | (0.000) | (0.000) | (0.000) | (0.000) | (0.000) |
| PC1 | -4.418** | -3.509 | -5.304* | -3.774* | -2.651 | -4.858* | -3.979* | -3.035 | -4.897* |
|  | (1.473) | (2.137) | (2.032) | (1.472) | (2.139) | (2.030) | (1.466) | (2.126) | (2.023) |
| PC2 | -3.720* | -2.080 | -5.211* | -3.759* | -2.207 | -5.162* | -3.717* | -2.068 | -5.218* |
|  | (1.451) | (2.134) | (2.047) | (1.458) | (2.164) | (2.048) | (1.451) | (2.140) | (2.047) |
| PC3 | 3.190* | 1.799 | 4.428* | 2.891* | 1.416 | 4.196* | 3.133* | 1.684 | 4.421* |
|  | (1.466) | (2.115) | (2.050) | (1.467) | (2.124) | (2.046) | (1.468) | (2.121) | (2.049) |
| Year dummies | Yes | Yes | Yes | Yes | Yes | Yes | Yes | Yes | Yes |
| Birth cohort dummies | Yes | Yes | Yes | Yes | Yes | Yes | Yes | Yes | Yes |
| Constant | 0.162 | 0.053 | 0.345 | 0.170 | 0.057 | 0.355 | 0.164 | 0.055 | 0.348 |
|  | (0.242) | (0.354) | (0.332) | (0.242) | (0.353) | (0.332) | (0.242) | (0.354) | (0.332) |
|  |  |  |  |  |  |  |  |  |  |
| Pseudo-R^2^ | 0.020 | 0.020 | 0.023 | 0.018 | 0.019 | 0.022 | 0.019 | 0.020 | 0.023 |
| p(χ^2^) | *** | *** | *** | *** | *** | *** | *** | *** | *** |

** P < 0.05, ** P < 0.005, *** P < 0.001; robust SE in parentheses; polygenic scores (PGS) for depression (DPGS) [21], bipolar disorder (BDPGS) [18], and mood disorders (MDPGS) [22].***Supplementary Table S22** Differences in predicted probabilities of economic satisfaction in the DPGS and MDPGS top three deciles compared to the lowest decile in the pooled 1992-2017 sample

| **Sample** | **All** | **Males** | **Females** | **All** | **Males** | **Females** |
| --- | --- | --- | --- | --- | --- | --- |
| **PGS** | **DPGS** | **DPGS** | **DPGS** | **MDPGS** | **MDPGS** | **MDPGS** |
| Total N in the model | 20,029 | 9,201 | 10,828 | 20,029 | 9,201 | 10,828 |
| **Reference: 1^st^ decile PGS** |  |  |  |  |  |  |
| Average marginal effects: |  |  |  |  |  |  |
| **8^th^ decile PGS** | -0.038*** | -0.028 | -0.044** | -0.038** | -0.028 | -0.044** |
|  | (0.012) | (0.017) | (0.016) | (0.012) | (0.017) | (0.016) |
| **9^th^ decile PGS** | -0.047*** | -0.043* | -0.050** | -0.042*** | -0.043* | -0.040* |
|  | (0.012) | (0.017) | (0.016) | (0.012) | (0.017) | (0.016) |
| **10^th^ decile PGS** | -0.053*** | -0.073*** | -0.037* | -0.051*** | -0.065*** | -0.037* |
|  | (0.012) | (0.018) | (0.016) | (0.012) | (0.018) | (0.016) |

** P < 0.05, ** P < 0.005, *** P < 0.001; robust SE in parentheses; polygenic scores (PGS) for depression (DPGS) [21], and mood disorders (MDPGS) [22]; table presenting average marginal effects from probit models of economic satisfaction with deciles of DPGS or MDPGS as main explanatory variable and controls for gender, age (linear and squared terms), birth cohort dummies, year dummies and PC1-3.*

**Supplementary Table S23** Coefficient estimates from probit regression models of economic satisfaction in the pooled 1992-2017 sample: Non-linear specification of polygenic scores

| **Sample** | **All** | **Males** | **Females** | **All** | **Males** | **Females** | **All** | **Males** | **Females** |
| --- | --- | --- | --- | --- | --- | --- | --- | --- | --- |
| **PGS** | **DPGS** | **DPGS** | **DPGS** | **BDPGS** | **BDPGS** | **BDPGS** | **MDPGS** | **MDPGS** | **MDPGS** |
| **PGS specification** | **Linear + squared** | **Linear + squared** | **Linear + squared** | **Linear + squared** | **Linear + squared** | **Linear + squared** | **Linear + squared** | **Linear + squared** | **Linear + squared** |
| N | 20,029 | 9,201 | 10,828 | 20,029 | 9,201 | 10,828 | 20,029 | 9,201 | 10,828 |
| Estimation | probit | probit | probit | probit | probit | probit | probit | probit | probit |
| **Dependent variable: Economic satisfaction** | | | | | | | | | |
| Explanatory variables: |  |  |  |  |  |  |  |  |  |
| PGS | -0.057*** | -0.057*** | -0.056*** | -0.014 | -0.004 | -0.023 | -0.053*** | -0.046** | -0.058*** |
|  | (0.011) | (0.015) | (0.015) | (0.011) | (0.015) | (0.015) | (0.011) | (0.015) | (0.015) |
| PGS squared | -0.005 | -0.020 | 0.007 | -0.006 | -0.014 | 0.001 | 0.003 | -0.003 | 0.010 |
|  | (0.007) | (0.010) | (0.010) | (0.007) | (0.011) | (0.010) | (0.008) | (0.011) | (0.011) |
| Female | 0.074*** |  |  | 0.073*** |  |  | 0.075*** |  |  |
|  | (0.021) |  |  | (0.021) |  |  | (0.021) |  |  |
| Age | 0.022* | 0.025 | 0.019 | 0.021* | 0.025 | 0.018 | 0.021* | 0.025 | 0.019 |
|  | (0.010) | (0.014) | (0.014) | (0.010) | (0.014) | (0.014) | (0.010) | (0.014) | (0.014) |
| Age squared | -0.000 | -0.000 | -0.000 | -0.000 | -0.000 | -0.000 | -0.000 | -0.000 | -0.000 |
|  | (0.000) | (0.000) | (0.000) | (0.000) | (0.000) | (0.000) | (0.000) | (0.000) | (0.000) |
| PC1 | -4.388** | -3.438 | -5.350* | -3.730* | -2.555 | -4.871* | -4.004* | -3.023 | -4.967* |
|  | (1.473) | (2.138) | (2.031) | (1.473) | (2.139) | (2.030) | (1.465) | (2.126) | (2.022) |
| PC2 | -3.723* | -2.062 | -5.201* | -3.714* | -2.082 | -5.166* | -3.707* | -2.065 | -5.187* |
|  | (1.452) | (2.134) | (2.048) | (1.457) | (2.154) | (2.048) | (1.451) | (2.140) | (2.047) |
| PC3 | 3.209* | 1.878 | 4.403* | 2.877* | 1.416 | 4.197* | 3.131* | 1.697 | 4.436* |
|  | (1.467) | (2.118) | (2.050) | (1.467) | (2.123) | (2.046) | (1.468) | (2.123) | (2.049) |
| Year dummies | Yes | Yes | Yes | Yes | Yes | Yes | Yes | Yes | Yes |
| Birth cohort dummies | Yes | Yes | Yes | Yes | Yes | Yes | Yes | Yes | Yes |
| Constant | 0.166 | 0.072 | 0.340 | 0.175 | 0.070 | 0.353 | 0.162 | 0.057 | 0.340 |
|  | (0.242) | (0.354) | (0.332) | (0.242) | (0.354) | (0.332) | (0.242) | (0.354) | (0.332) |
|  |  |  |  |  |  |  |  |  |  |
| Pseudo-R^2^ | 0.020 | 0.021 | 0.023 | 0.018 | 0.019 | 0.022 | 0.019 | 0.020 | 0.023 |
| p(χ^2^) | *** | *** | *** | *** | *** | *** | *** | *** | *** |

** P < 0.05, ** P < 0.005, *** P < 0.001; robust SE in parentheses; polygenic scores (PGS) for depression (DPGS) [21], bipolar disorder (BDPGS) [18], and mood disorders (MDPGS) [22].*

**Supplementary Table S24** Coefficient estimates from probit regression models of economic satisfaction within the lowest equivalent income subsample in the pooled 1992-2017 sample

| **Sample** | **All** | **Males** | **Females** | **All** | **Males** | **Females** | **All** | **Males** | **Females** |
| --- | --- | --- | --- | --- | --- | --- | --- | --- | --- |
| **PGS** | **DPGS** | **DPGS** | **DPGS** | **BDPGS** | **BDPGS** | **BDPGS** | **MDPGS** | **MDPGS** | **MDPGS** |
| **PGS specification** | **Linear** | **Linear** | **Linear** | **Linear** | **Linear** | **Linear** | **Linear** | **Linear** | **Linear** |
| N | 6,489 | 2,881 | 3,608 | 6,489 | 2,881 | 3,608 | 6,489 | 2,881 | 3,608 |
| Estimation | probit | probit | probit | probit | probit | probit | probit | probit | probit |
| **Dependent variable: Economic satisfaction** | | | | | | | | | |
| Explanatory variables: |  |  |  |  |  |  |  |  |  |
| PGS | -0.070** | -0.074** | -0.035 | -0.015 | -0.016 | -0.025 | -0.044 | -0.047 | -0.034 |
|  | (0.024) | (0.025) | (0.023) | (0.024) | (0.025) | (0.022) | (0.024) | (0.024) | (0.022) |
| Female | 0.121*** |  |  | 0.122*** |  |  | 0.121*** |  |  |
|  | (0.033) |  |  | (0.033) |  |  | (0.033) |  |  |
| PGS # Female | 0.033 |  |  | -0.013 |  |  | 0.008 |  |  |
|  | (0.033) |  |  | (0.033) |  |  | (0.033) |  |  |
| Age | -0.025 | -0.031 | -0.019 | -0.026 | -0.031 | -0.019 | -0.025 | -0.031 | -0.018 |
|  | (0.015) | (0.022) | (0.020) | (0.015) | (0.022) | (0.020) | (0.015) | (0.022) | (0.020) |
| Age squared | 0.000* | 0.000 | 0.000 | 0.000* | 0.000 | 0.000 | 0.000* | 0.000 | 0.000 |
|  | (0.000) | (0.000) | (0.000) | (0.000) | (0.000) | (0.000) | (0.000) | (0.000) | (0.000) |
| PC1 | -8.010*** | -9.499** | -6.624* | -7.666*** | -8.766* | -6.568* | -7.601*** | -8.879* | -6.381* |
|  | (2.246) | (3.304) | (3.077) | (2.248) | (3.305) | (3.080) | (2.239) | (3.293) | (3.068) |
| PC2 | -0.462 | 1.113 | -1.902 | -0.535 | 0.869 | -1.842 | -0.552 | 1.013 | -1.987 |
|  | (2.083) | (2.899) | (3.009) | (2.083) | (2.902) | (3.010) | (2.082) | (2.898) | (3.009) |
| PC3 | 7.333*** | 6.837* | 8.067* | 6.994*** | 6.176* | 7.962* | 7.181*** | 6.558* | 8.013* |
|  | (2.106) | (3.023) | (2.960) | (2.103) | (3.022) | (2.955) | (2.106) | (3.027) | (2.959) |
| Year dummies | Yes | Yes | Yes | Yes | Yes | Yes | Yes | Yes | Yes |
| Birth cohort dummies | Yes | Yes | Yes | Yes | Yes | Yes | Yes | Yes | Yes |
| Constant | 0.708 | 0.829 | 0.690 | 0.712 | 0.827 | 0.700 | 0.703 | 0.827 | 0.686 |
|  | (0.366) | (0.551) | (0.491) | (0.365) | (0.549) | (0.492) | (0.366) | (0.549) | (0.491) |
|  |  |  |  |  |  |  |  |  |  |
| Pseudo-R^2^ | 0.033 | 0.036 | 0.035 | 0.032 | 0.033 | 0.034 | 0.032 | 0.034 | 0.035 |
| p(χ^2^) | *** | *** | *** | *** | *** | *** | *** | *** | *** |

** P < 0.05, ** P < 0.005, *** P < 0.001; robust SE in parentheses; polygenic scores (PGS) for depression (DPGS) [21], bipolar disorder (BDPGS) [18], and mood disorders (MDPGS) [22].*

**Supplementary Table S25** Coefficient estimates from probit regression models of economic satisfaction within the medium equivalent income subsample in the pooled 1992-2017 sample

| **Sample** | **All** | **Males** | **Females** | **All** | **Males** | **Females** | **All** | **Males** | **Females** |
| --- | --- | --- | --- | --- | --- | --- | --- | --- | --- |
| **PGS** | **DPGS** | **DPGS** | **DPGS** | **BDPGS** | **BDPGS** | **BDPGS** | **MDPGS** | **MDPGS** | **MDPGS** |
| **PGS specification** | **Linear** | **Linear** | **Linear** | **Linear** | **Linear** | **Linear** | **Linear** | **Linear** | **Linear** |
| N | 6,858 | 3,056 | 3,802 | 6,858 | 3,056 | 3,802 | 6,858 | 3,056 | 3,802 |
| Estimation | probit | probit | probit | probit | probit | probit | probit | probit | probit |
| **Dependent variable: Economic satisfaction** | | | | | | | | | |
| Explanatory variables: |  |  |  |  |  |  |  |  |  |
| PGS | -0.014 | -0.008 | -0.052 | 0.024 | 0.026 | -0.052* | -0.019 | -0.015 | -0.074* |
|  | (0.029) | (0.029) | (0.027) | (0.028) | (0.028) | (0.026) | (0.028) | (0.028) | (0.027) |
| Female | 0.094* |  |  | 0.092* |  |  | 0.096* |  |  |
|  | (0.038) |  |  | (0.038) |  |  | (0.038) |  |  |
| PGS # Female | -0.038 |  |  | -0.073 |  |  | -0.054 |  |  |
|  | (0.039) |  |  | (0.038) |  |  | (0.039) |  |  |
| Age | -0.027 | -0.033 | -0.023 | -0.027 | -0.033 | -0.024 | -0.027 | -0.034 | -0.024 |
|  | (0.018) | (0.026) | (0.025) | (0.018) | (0.027) | (0.025) | (0.018) | (0.026) | (0.025) |
| Age squared | 0.001* | 0.001 | 0.001* | 0.001** | 0.001 | 0.001* | 0.001** | 0.001 | 0.001* |
|  | (0.000) | (0.000) | (0.000) | (0.000) | (0.000) | (0.000) | (0.000) | (0.000) | (0.000) |
| PC1 | -13.539*** | -12.856** | -14.420*** | -13.286*** | -12.330** | -14.512*** | -13.427*** | -12.861*** | -14.260*** |
|  | (2.683) | (3.918) | (3.690) | (2.682) | (3.909) | (3.697) | (2.670) | (3.896) | (3.676) |
| PC2 | -7.539* | -5.974 | -8.966* | -7.424* | -6.124 | -8.588* | -7.442* | -5.952 | -8.801* |
|  | (2.742) | (4.023) | (3.947) | (2.752) | (4.061) | (3.956) | (2.745) | (4.019) | (3.958) |
| PC3 | 4.491 | 2.100 | 6.481 | 4.464 | 2.001 | 6.522 | 4.607 | 2.146 | 6.657 |
|  | (2.723) | (3.979) | (3.766) | (2.726) | (3.988) | (3.767) | (2.723) | (3.978) | (3.768) |
| Year dummies | Yes | Yes | Yes | Yes | Yes | Yes | Yes | Yes | Yes |
| Birth cohort dummies | Yes | Yes | Yes | Yes | Yes | Yes | Yes | Yes | Yes |
| Constant | 1.174* | 1.361* | 1.161 | 1.191* | 1.354* | 1.195* | 1.191* | 1.364* | 1.190* |
|  | (0.439) | (0.647) | (0.595) | (0.440) | (0.648) | (0.596) | (0.440) | (0.648) | (0.595) |
|  |  |  |  |  |  |  |  |  |  |
| Pseudo-R^2^ | 0.030 | 0.028 | 0.036 | 0.030 | 0.029 | 0.036 | 0.031 | 0.028 | 0.037 |
| p(χ^2^) | *** | *** | *** | *** | *** | *** | *** | *** | *** |

** P < 0.05, ** P < 0.005, *** P < 0.001; robust SE in parentheses; polygenic scores (PGS) for depression (DPGS) [21], bipolar disorder (BDPGS) [18], and mood disorders (MDPGS) [22].*

**Supplementary Table S26** Coefficient estimates from probit regression models of economic satisfaction within the highest equivalent income subsample in the pooled 1992-2017 sample

| **Sample** | **All** | **Males** | **Females** | **All** | **Males** | **Females** | **All** | **Males** | **Females** |
| --- | --- | --- | --- | --- | --- | --- | --- | --- | --- |
| **PGS** | **DPGS** | **DPGS** | **DPGS** | **BDPGS** | **BDPGS** | **BDPGS** | **MDPGS** | **MDPGS** | **MDPGS** |
| **PGS specification** | **Linear** | **Linear** | **Linear** | **Linear** | **Linear** | **Linear** | **Linear** | **Linear** | **Linear** |
| N | 6,270 | 3,126 | 3,144 | 6,270 | 3,126 | 3,144 | 6,270 | 3,126 | 3,144 |
| Estimation | probit | probit | probit | probit | probit | probit | probit | probit | probit |
| **Dependent variable: Economic satisfaction** | | | | | | | | | |
| Explanatory variables: |  |  |  |  |  |  |  |  |  |
| PGS | -0.055 | -0.050 | -0.069 | 0.026 | 0.033 | 0.011 | -0.038 | -0.033 | -0.075* |
|  | (0.034) | (0.034) | (0.036) | (0.036) | (0.036) | (0.036) | (0.035) | (0.035) | (0.032) |
| Female | 0.096 |  |  | 0.093 |  |  | 0.098 |  |  |
|  | (0.051) |  |  | (0.051) |  |  | (0.051) |  |  |
| PGS # Female | -0.011 |  |  | -0.016 |  |  | -0.036 |  |  |
|  | (0.049) |  |  | (0.051) |  |  | (0.048) |  |  |
| Age | -0.006 | 0.012 | -0.027 | -0.008 | 0.010 | -0.027 | -0.006 | 0.012 | -0.025 |
|  | (0.027) | (0.038) | (0.038) | (0.027) | (0.038) | (0.038) | (0.027) | (0.038) | (0.038) |
| Age squared | 0.000 | 0.000 | 0.000 | 0.000 | 0.000 | 0.001 | 0.000 | 0.000 | 0.000 |
|  | (0.000) | (0.000) | (0.000) | (0.000) | (0.000) | (0.000) | (0.000) | (0.000) | (0.000) |
| PC1 | -7.472 | -4.276 | -11.353* | -6.017 | -2.601 | -9.949 | -6.957 | -3.835 | -10.709* |
|  | (3.838) | (5.463) | (5.337) | (3.792) | (5.415) | (5.254) | (3.805) | (5.424) | (5.290) |
| PC2 | -1.167 | 3.879 | -5.740 | -1.119 | 4.312 | -6.008 | -1.109 | 4.107 | -5.834 |
|  | (3.794) | (5.713) | (5.499) | (3.852) | (5.823) | (5.526) | (3.829) | (5.751) | (5.549) |
| PC3 | 0.937 | -2.467 | 4.494 | 0.391 | -3.115 | 3.968 | 0.779 | -2.804 | 4.529 |
|  | (3.764) | (5.451) | (5.253) | (3.782) | (5.525) | (5.220) | (3.774) | (5.498) | (5.225) |
| Year dummies | Yes | Yes | Yes | Yes | Yes | Yes | Yes | Yes | Yes |
| Birth cohort dummies | Yes | Yes | Yes | Yes | Yes | Yes | Yes | Yes | Yes |
| Constant | 1.063 | 0.529 | 1.757 | 1.084 | 0.566 | 1.753 | 1.042 | 0.518 | 1.725 |
|  | (0.653) | (0.937) | (0.916) | (0.650) | (0.934) | (0.912) | (0.653) | (0.937) | (0.917) |
|  |  |  |  |  |  |  |  |  |  |
| Pseudo-R^2^ | 0.028 | 0.039 | 0.027 | 0.027 | 0.038 | 0.024 | 0.028 | 0.038 | 0.027 |
| p(χ^2^) | *** | *** | ** | *** | *** | * | *** | *** | ** |

** P < 0.05, ** P < 0.005, *** P < 0.001; robust SE in parentheses; polygenic scores (PGS) for depression (DPGS) [21], bipolar disorder (BDPGS) [18], and mood disorders (MDPGS) [22].*

**Supplementary Table S27** Sensitivity analysis of coefficient estimates from ordered probit models of education in the pooled 1992-2017 sample, including and excluding participants with diagnosed mental disorders

| **Sample** | **All** | **All with no diagnosed mental disorders** | **All** | **All with no diagnosed mental disorders** | **All** | **All with no diagnosed mental disorders** |
| --- | --- | --- | --- | --- | --- | --- |
| **PGS** | **DPGS** | **DPGS** | **BDPGS** | **BDPGS** | **MDPGS** | **MDPGS** |
| PGS specification | linear | linear | linear | linear | linear | linear |
| N | 20,121 | 18,158 | 20,121 | 18,158 | 20,121 | 18,158 |
| Model | oprobit | oprobit | oprobit | oprobit | oprobit | oprobit |
| **Dependent variable: Education** |  |  |  |  |  |  |
| **Explanatory variables:** |  |  |  |  |  |  |
| PGS | -0.044*** | -0.039** | 0.027* | 0.036** | -0.003 | 0.003 |
|  | (0.012) | (0.013) | (0.012) | (0.013) | (0.012) | (0.013) |
| Female | 0.196*** | 0.183*** | 0.195*** | 0.181*** | 0.196*** | 0.181*** |
|  | (0.016) | (0.017) | (0.016) | (0.017) | (0.016) | (0.017) |
| PGS # Female | 0.010 | 0.006 | -0.006 | -0.009 | -0.012 | -0.017 |
|  | (0.016) | (0.017) | (0.016) | (0.017) | (0.016) | (0.017) |
| Birth cohort 1920-1930ies | -0.976*** | -0.999*** | -0.976*** | -0.998*** | -0.976*** | -0.998*** |
|  | (0.035) | (0.037) | (0.035) | (0.037) | (0.035) | (0.037) |
| Birth cohort 1940ies | -0.441*** | -0.461*** | -0.440*** | -0.461*** | -0.440*** | -0.460*** |
|  | (0.027) | (0.028) | (0.027) | (0.028) | (0.027) | (0.028) |
| Birth cohort 1950ies | Reference | Reference | Reference | Reference | Reference | Reference |
| Birth cohort 1960ies | 0.263*** | 0.255*** | 0.262*** | 0.255*** | 0.263*** | 0.256*** |
|  | (0.022) | (0.023) | (0.022) | (0.023) | (0.022) | (0.023) |
| Birth cohort 1970ies | 0.398*** | 0.387*** | 0.399*** | 0.388*** | 0.399*** | 0.388*** |
|  | (0.024) | (0.026) | (0.024) | (0.026) | (0.024) | (0.026) |
| Birth cohort 1980-1990ies | 0.374*** | 0.369*** | 0.376*** | 0.371*** | 0.375*** | 0.370*** |
|  | (0.029) | (0.032) | (0.030) | (0.032) | (0.029) | (0.032) |
| PC1 | 8.526*** | 8.712*** | 9.456*** | 9.711*** | 9.035*** | 9.200*** |
|  | (1.149) | (1.212) | (1.150) | (1.213) | (1.145) | (1.208) |
| PC2 | -4.134*** | -4.389*** | -4.305*** | -4.580*** | -4.183*** | -4.432*** |
|  | (1.164) | (1.217) | (1.167) | (1.225) | (1.163) | (1.218) |
| PC3 | 0.147 | 0.874 | -0.116 | 0.606 | -0.011 | 0.712 |
|  | (1.098) | (1.164) | (1.099) | (1.165) | (1.098) | (1.164) |
| Cut: Primary/Secondary education | -0.824*** | -0.864*** | -0.825*** | -0.866*** | -0.824*** | -0.864*** |
|  | (0.019) | (0.020) | (0.019) | (0.020) | (0.019) | (0.020) |
| Cut: Secondary/Higher education | 0.445*** | 0.404*** | 0.444*** | 0.402*** | 0.445*** | 0.403*** |
|  | (0.018) | (0.020) | (0.018) | (0.020) | (0.018) | (0.020) |
|  |  |  |  |  |  |  |
| Pseudo-R^2^ | 0.071 | 0.072 | 0.070 | 0.072 | 0.070 | 0.072 |
| p(χ^2^) | *** | *** | *** | *** | *** | *** |

** P < 0.05, ** P < 0.005, *** P < 0.001; robust SE in parentheses; DPGS denotes Depression Polygenic Score [21], BDPGS Bipolar Disorder Polygenic Score [18] and MDPGS Mood Disorders Polygenic Score [22].*

**Supplementary Table S28** Sensitivity analysis of economic outcomes in the pooled 1992–2017 sample using PC1–10 as genetic controls: Depression Polygenic Score (DPGS)

| **Sample** | **All** | **All** | **All** | **All** | **All** | **All** | **All** | **All** |
| --- | --- | --- | --- | --- | --- | --- | --- | --- |
| **PGS** | **DPGS** | **DPGS** | **DPGS** | **DPGS** | **DPGS** | **DPGS** | **DPGS** | **DPGS** |
| **PGS specification** | **Linear** | **Linear** | **Linear** | **Linear** | **Linear** | **Linear** | **Linear** | **Linear** |
| N | 20,121 | 19,707 | 19,707 | 19,707 | 19,707 | 19,707 | 19,685 | 20,029 |
| Model | oprobit | probit | probit | probit | probit | probit | oprobit | probit |
| **Dependent variable:** | **Education** | **Non-employed** | **Self-employed** | **Physical**  **work** | **Office**  **work** | **Knowledge work** | **Equivalent income** | **Economic satisfaction** |
| **Explanatory variables:** |  |  |  |  |  |  |  |  |
| PGS | -0.043*** | 0.064*** | -0.058*** | 0.010 | -0.017 | -0.029 | -0.036** | -0.059*** |
|  | (0.012) | (0.015) | (0.017) | (0.014) | (0.016) | (0.016) | (0.012) | (0.016) |
| Female | 0.194*** | -0.107*** | -0.291*** | -0.424*** | 0.656*** | -0.039 | -0.092*** | 0.074*** |
|  | (0.016) | (0.021) | (0.026) | (0.021) | (0.020) | (0.022) | (0.016) | (0.021) |
| PGS # Female | 0.010 | 0.010 | 0.040 | -0.024 | 0.021 | -0.035 | -0.001 | 0.003 |
|  | (0.016) | (0.021) | (0.025) | (0.021) | (0.020) | (0.021) | (0.016) | (0.021) |
| Age |  | -0.257*** | 0.123*** | 0.048*** | 0.066*** | 0.128*** | 0.112*** | 0.021* |
|  |  | (0.010) | (0.013) | (0.010) | (0.009) | (0.010) | (0.007) | (0.010) |
| Age squared |  | 0.003*** | -0.001*** | -0.001*** | -0.001*** | -0.002*** | -0.001*** | -0.000 |
|  |  | (0.000) | (0.000) | (0.000) | (0.000) | (0.000) | (0.000) | (0.000) |
| PC1 | 9.123*** | -11.876*** | -4.083* | -3.066* | 2.230 | 16.755*** | 13.674*** | -4.238** |
|  | (1.150) | (1.512) | (1.802) | (1.434) | (1.387) | (1.530) | (1.165) | (1.479) |
| PC2 | -4.774*** | 0.732 | -0.437 | 2.091 | -0.830 | -2.246 | -3.217* | -3.963* |
|  | (1.175) | (1.692) | (1.767) | (1.483) | (1.405) | (1.456) | (1.195) | (1.472) |
| PC3 | 0.298 | 3.630* | -1.898 | -0.507 | 0.209 | -2.289 | -3.444** | 3.239* |
|  | (1.095) | (1.479) | (1.754) | (1.448) | (1.334) | (1.414) | (1.121) | (1.484) |
| PC4 | 4.424*** | -1.102 | -6.191*** | -1.364 | 2.445 | 3.651* | 7.860*** | -0.635 |
|  | (1.173) | (1.536) | (1.698) | (1.429) | (1.346) | (1.441) | (1.156) | (1.436) |
| PC5 | -4.872*** | 0.369 | 0.584 | 4.023* | -0.048 | -5.511*** | -4.471*** | -2.430 |
|  | (1.153) | (1.561) | (1.917) | (1.541) | (1.445) | (1.570) | (1.231) | (1.587) |
| PC6 | 3.364** | -1.374 | -2.650 | -2.887* | 0.786 | 5.127*** | 3.638** | 0.360 |
|  | (1.119) | (1.464) | (1.870) | (1.450) | (1.334) | (1.396) | (1.120) | (1.459) |
| PC7 | -2.741* | 1.420 | -1.957 | 2.201 | 0.458 | -2.021 | 0.885 | -0.651 |
|  | (1.112) | (1.510) | (1.788) | (1.437) | (1.360) | (1.442) | (1.137) | (1.493) |
| PC8 | -2.253* | 1.115 | 0.992 | 1.271 | -1.673 | -1.693 | -0.865 | -0.643 |
|  | (1.105) | (1.497) | (1.814) | (1.454) | (1.366) | (1.451) | (1.150) | (1.491) |
| PC9 | 0.830 | -0.102 | 0.518 | -2.270 | 0.768 | 1.095 | 2.596* | -0.227 |
|  | (1.125) | (1.504) | (1.829) | (1.489) | (1.409) | (1.530) | (1.161) | (1.549) |
| PC10 | -4.784*** | -1.652 | 6.480*** | 4.444** | 0.589 | -7.978*** | -3.954*** | 2.656 |
|  | (1.117) | (1.479) | (1.762) | (1.439) | (1.363) | (1.483) | (1.140) | (1.480) |
| Year dummies | No | Yes | Yes | Yes | Yes | Yes | Yes | Yes |
| Birth cohort dummies | Yes | Yes | Yes | Yes | Yes | Yes | Yes | Yes |
| Constant |  | 3.709*** | -3.910*** | -1.173*** | -1.762*** | -3.575*** |  | 0.168 |
|  |  | (0.241) | (0.313) | (0.238) | (0.226) | (0.251) |  | (0.242) |
| Cut: Categories 1/2 | -0.830*** |  |  |  |  |  | 1.720*** |  |
|  | (0.019) |  |  |  |  |  | (0.184) |  |
| Cut: Categories 2/3 | 0.443*** |  |  |  |  |  | 2.656*** |  |
|  | (0.018) |  |  |  |  |  | (0.185) |  |
|  |  |  |  |  |  |  |  |  |
| Pseudo-R^2^ | 0.072 | 0.131 | 0.031 | 0.036 | 0.067 | 0.040 | 0.024 | 0.020 |
| p(χ^2^) | *** | *** | *** | *** | *** | *** | *** | *** |

** P < 0.05, ** P < 0.005, *** P < 0.001; robust SE in parentheses; polygenic score (PGS) for depression (DPGS) [21]*

**Supplementary Table S29** Sensitivity analysis of economic outcomes in the pooled 1992–2017 sample using PC1–10 as genetic controls: Bipolar Disorder Polygenic Score (BDPGS)

| **Sample** | **All** | **All** | **All** | **All** | **All** | **All** | **All** | **All** |
| --- | --- | --- | --- | --- | --- | --- | --- | --- |
| **PGS** | **BDPGS** | **BDPGS** | **BDPGS** | **BDPGS** | **BDPGS** | **BDPGS** | **BDPGS** | **BDPGS** |
| **PGS specification** | **Linear** | **Linear** | **Linear** | **Linear** | **Linear** | **Linear** | **Linear** | **Linear** |
| N | 20,121 | 19,707 | 19,707 | 19,707 | 19,707 | 19,707 | 19,685 | 20,029 |
| Model | oprobit | probit | probit | probit | probit | probit | oprobit | probit |
| **Dependent variable:** | **Education** | **Non-employed** | **Self-employed** | **Physical**  **work** | **Office**  **work** | **Knowledge work** | **Equivalent income** | **Economic satisfaction** |
| **Explanatory variables:** |  |  |  |  |  |  |  |  |
| PGS | 0.028* | 0.055*** | -0.019 | -0.063*** | -0.008 | 0.040* | -0.016 | -0.004 |
|  | (0.012) | (0.015) | (0.017) | (0.014) | (0.016) | (0.016) | (0.012) | (0.015) |
| Female | 0.193*** | -0.106*** | -0.293*** | -0.422*** | 0.656*** | -0.040 | -0.093*** | 0.073*** |
|  | (0.016) | (0.021) | (0.026) | (0.021) | (0.020) | (0.022) | (0.016) | (0.021) |
| PGS # Female | -0.006 | -0.010 | 0.026 | 0.023 | -0.031 | 0.012 | 0.020 | -0.019 |
|  | (0.016) | (0.021) | (0.026) | (0.020) | (0.020) | (0.021) | (0.016) | (0.021) |
| Age |  | -0.256*** | 0.123*** | 0.048*** | 0.066*** | 0.129*** | 0.112*** | 0.021* |
|  |  | (0.010) | (0.013) | (0.010) | (0.009) | (0.010) | (0.007) | (0.010) |
| Age squared |  | 0.003*** | -0.001*** | -0.001*** | -0.001*** | -0.002*** | -0.001*** | -0.000 |
|  |  | (0.000) | (0.000) | (0.000) | (0.000) | (0.000) | (0.000) | (0.000) |
| PC1 | 10.030*** | -12.146*** | -3.592* | -3.844* | 1.924 | 18.098*** | 14.128*** | -3.607* |
|  | (1.149) | (1.510) | (1.804) | (1.433) | (1.386) | (1.535) | (1.164) | (1.478) |
| PC2 | -4.944*** | 0.557 | -0.472 | 2.333 | -0.737 | -2.524 | -3.238* | -3.985* |
|  | (1.174) | (1.695) | (1.758) | (1.493) | (1.406) | (1.447) | (1.196) | (1.479) |
| PC3 | 0.048 | 3.997* | -2.060 | -0.435 | 0.262 | -2.652 | -3.637** | 2.926* |
|  | (1.093) | (1.479) | (1.752) | (1.446) | (1.333) | (1.415) | (1.121) | (1.485) |
| PC4 | 4.720*** | -0.725 | -6.126*** | -1.808 | 2.293 | 4.036** | 7.849*** | -0.597 |
|  | (1.172) | (1.542) | (1.694) | (1.440) | (1.349) | (1.437) | (1.158) | (1.446) |
| PC5 | -4.725*** | 0.472 | 0.569 | 3.818* | -0.133 | -5.264*** | -4.460*** | -2.383 |
|  | (1.151) | (1.566) | (1.919) | (1.542) | (1.446) | (1.570) | (1.232) | (1.591) |
| PC6 | 3.258** | -1.504 | -2.655 | -2.692 | 0.920 | 4.973*** | 3.629** | 0.454 |
|  | (1.117) | (1.464) | (1.869) | (1.452) | (1.335) | (1.393) | (1.120) | (1.461) |
| PC7 | -2.806* | 1.329 | -1.938 | 2.311 | 0.556 | -2.193 | 0.874 | -0.633 |
|  | (1.112) | (1.512) | (1.784) | (1.440) | (1.359) | (1.441) | (1.138) | (1.496) |
| PC8 | -2.358* | 1.209 | 0.916 | 1.317 | -1.688 | -1.757 | -0.917 | -0.785 |
|  | (1.103) | (1.498) | (1.811) | (1.455) | (1.364) | (1.448) | (1.150) | (1.494) |
| PC9 | 1.261 | -0.090 | 0.678 | -2.768 | 0.565 | 1.746 | 2.759* | -0.042 |
|  | (1.125) | (1.506) | (1.828) | (1.490) | (1.411) | (1.532) | (1.162) | (1.552) |
| PC10 | -4.690*** | -1.551 | 6.495*** | 4.308** | 0.562 | -7.941*** | -3.951*** | 2.701 |
|  | (1.117) | (1.478) | (1.762) | (1.439) | (1.362) | (1.481) | (1.141) | (1.481) |
| Year dummies | No | Yes | Yes | Yes | Yes | Yes | Yes | Yes |
| Birth cohort dummies | Yes | Yes | Yes | Yes | Yes | Yes | Yes | Yes |
| Constant |  | 3.689*** | -3.907*** | -1.174*** | -1.756*** | -3.579*** |  | 0.176 |
|  |  | (0.241) | (0.313) | (0.238) | (0.227) | (0.251) |  | (0.242) |
| Cut: Categories 1/2 | -0.830*** |  |  |  |  |  | 1.716*** |  |
|  | (0.019) |  |  |  |  |  | (0.184) |  |
| Cut: Categories 2/3 | 0.442*** |  |  |  |  |  | 2.652*** |  |
|  | (0.018) |  |  |  |  |  | (0.185) |  |
|  |  |  |  |  |  |  |  |  |
| Pseudo-R^2^ | 0.072 | 0.130 | 0.030 | 0.037 | 0.068 | 0.040 | 0.023 | 0.018 |
| p(χ^2^) | *** | *** | *** | *** | *** | *** | *** | *** |

** P < 0.05, ** P < 0.005, *** P < 0.001; robust SE in parentheses; polygenic score (PGS) for bipolar disorder (BDPGS) [18]*

**Supplementary Table S30** Sensitivity analysis of economic outcomes in the pooled 1992–2017 sample using PC1–10 as genetic controls: Mood Disorders Polygenic Score (MDPGS)

| **Sample** | **All** | **All** | **All** | **All** | **All** | **All** | **All** | **All** |
| --- | --- | --- | --- | --- | --- | --- | --- | --- |
| **PGS** | **MDPGS** | **MDPGS** | **MDPGS** | **MDPGS** | **MDPGS** | **MDPGS** | **MDPGS** | **MDPGS** |
| **PGS specification** | **Linear** | **Linear** | **Linear** | **Linear** | **Linear** | **Linear** | **Linear** | **Linear** |
| N | 20,121 | 19,707 | 19,707 | 19,707 | 19,707 | 19,707 | 19,685 | 20,029 |
| Model | oprobit | probit | probit | probit | probit | probit | oprobit | probit |
| **Dependent variable:** | **Education** | **Non-employed** | **Self-employed** | **Physical**  **work** | **Office**  **work** | **Knowledge work** | **Equivalent income** | **Economic satisfaction** |
| **Explanatory variables:** |  |  |  |  |  |  |  |  |
| PGS | -0.003 | 0.069*** | -0.056** | -0.024 | -0.003 | -0.003 | -0.035** | -0.047** |
|  | (0.012) | (0.015) | (0.017) | (0.014) | (0.016) | (0.016) | (0.012) | (0.015) |
| Female | 0.194*** | -0.107*** | -0.291*** | -0.423*** | 0.656*** | -0.038 | -0.092*** | 0.075*** |
|  | (0.016) | (0.021) | (0.026) | (0.021) | (0.020) | (0.022) | (0.016) | (0.021) |
| PGS # Female | -0.013 | 0.005 | 0.054* | 0.011 | -0.025 | -0.020 | 0.008 | -0.011 |
|  | (0.016) | (0.021) | (0.026) | (0.021) | (0.020) | (0.021) | (0.016) | (0.021) |
| Age |  | -0.256*** | 0.123*** | 0.048*** | 0.066*** | 0.128*** | 0.112*** | 0.021* |
|  |  | (0.010) | (0.013) | (0.010) | (0.009) | (0.010) | (0.007) | (0.010) |
| Age squared |  | 0.003*** | -0.001*** | -0.001*** | -0.001*** | -0.002*** | -0.001*** | -0.000 |
|  |  | (0.000) | (0.000) | (0.000) | (0.000) | (0.000) | (0.000) | (0.000) |
| PC1 | 9.618*** | -12.354*** | -3.773* | -3.220* | 2.193 | 17.362*** | 13.975*** | -3.790* |
|  | (1.146) | (1.504) | (1.796) | (1.427) | (1.382) | (1.527) | (1.160) | (1.471) |
| PC2 | -4.819*** | 0.707 | -0.412 | 2.122 | -0.836 | -2.286 | -3.211* | -3.951* |
|  | (1.172) | (1.691) | (1.763) | (1.486) | (1.405) | (1.451) | (1.194) | (1.472) |
| PC3 | 0.141 | 3.661* | -1.922 | -0.425 | 0.284 | -2.483 | -3.488** | 3.165* |
|  | (1.094) | (1.480) | (1.751) | (1.447) | (1.334) | (1.412) | (1.121) | (1.487) |
| PC4 | 4.471*** | -0.943 | -6.215*** | -1.463 | 2.436 | 3.647* | 7.804*** | -0.706 |
|  | (1.170) | (1.537) | (1.697) | (1.432) | (1.346) | (1.438) | (1.155) | (1.438) |
| PC5 | -4.842*** | 0.415 | 0.557 | 4.000* | -0.086 | -5.458*** | -4.485*** | -2.461 |
|  | (1.151) | (1.562) | (1.917) | (1.541) | (1.445) | (1.568) | (1.231) | (1.590) |
| PC6 | 3.410** | -1.674 | -2.539 | -2.787 | 0.893 | 5.203*** | 3.776*** | 0.640 |
|  | (1.119) | (1.467) | (1.868) | (1.451) | (1.335) | (1.394) | (1.121) | (1.461) |
| PC7 | -2.724* | 1.349 | -1.977 | 2.207 | 0.524 | -2.026 | 0.912 | -0.596 |
|  | (1.111) | (1.510) | (1.788) | (1.438) | (1.359) | (1.441) | (1.138) | (1.494) |
| PC8 | -2.328* | 1.129 | 0.976 | 1.310 | -1.663 | -1.766 | -0.882 | -0.691 |
|  | (1.103) | (1.497) | (1.810) | (1.454) | (1.365) | (1.449) | (1.150) | (1.493) |
| PC9 | 0.987 | -0.042 | 0.573 | -2.422 | 0.693 | 1.229 | 2.592* | -0.263 |
|  | (1.125) | (1.505) | (1.832) | (1.489) | (1.409) | (1.529) | (1.161) | (1.550) |
| PC10 | -4.746*** | -1.576 | 6.460*** | 4.400** | 0.607 | -7.949*** | -3.963*** | 2.642 |
|  | (1.117) | (1.479) | (1.762) | (1.439) | (1.362) | (1.482) | (1.140) | (1.482) |
| Year dummies | No | Yes | Yes | Yes | Yes | Yes | Yes | Yes |
| Birth cohort dummies | Yes | Yes | Yes | Yes | Yes | Yes | Yes | Yes |
| Constant |  | 3.709*** | -3.907*** | -1.174*** | -1.762*** | -3.576*** |  | 0.170 |
|  |  | (0.241) | (0.313) | (0.238) | (0.227) | (0.251) |  | (0.242) |
| Cut: Categories 1/2 | -0.829*** |  |  |  |  |  | 1.718*** |  |
|  | (0.019) |  |  |  |  |  | (0.184) |  |
| Cut: Categories 2/3 | 0.442*** |  |  |  |  |  | 2.654*** |  |
|  | (0.018) |  |  |  |  |  | (0.185) |  |
|  |  |  |  |  |  |  |  |  |
| Pseudo-R^2^ | 0.072 | 0.131 | 0.031 | 0.036 | 0.067 | 0.039 | 0.024 | 0.020 |
| p(χ^2^) | *** | *** | *** | *** | *** | *** | *** | *** |

** P < 0.05, ** P < 0.005, *** P < 0.001; robust SE in parentheses; polygenic score (PGS) for mood disorders (MDPGS) [18]*
